# Highly Diverse Aquatic Microbial Communities Separated by Permafrost in Greenland Show Distinct Features According to Environmental Niches

**DOI:** 10.3389/fmicb.2019.01583

**Published:** 2019-07-11

**Authors:** Malin Bomberg, Lillemor Claesson Liljedahl, Tiina Lamminmäki, Anne Kontula

**Affiliations:** ^1^VTT Technical Research Centre of Finland Ltd., Espoo, Finland; ^2^Svensk Kärnbränslehantering AB, Solna, Sweden; ^3^Posiva Oy, Eurajoki, Finland

**Keywords:** deep biosphere, nitrogen fixation, iron reduction, photosynthesis, groundwater, metabolic profile

## Abstract

The Greenland Analog Project (GAP) study area in the vicinity of Kangarlussuaq, Western Greenland, was sampled for surface water and deep groundwater in order to determine the composition and estimate the metabolic features of the microbial communities in water bodies separated by permafrost. The sampling sites comprised a freshwater pond, talik lake, deep anoxic groundwater, glacier ice and supraglacial river, meltwater river and melting permafrost active layer. The microbial communities were characterized by amplicon sequencing of the bacterial and archaeal 16S rRNA genes and fungal ITS1 spacer. In addition, bacterial, archaeal and fungal numbers were determined by qPCR and plate counts, and the utilization pattern of carbon and nitrogen substrates was determined with Biolog AN plates and metabolic functions were predicted with FAPROTAX. Different sample types were clearly distinguishable from each other based on community composition, microbial numbers, and substrate utilization patterns, forming four groups, (1) pond/lake, (2) deep groundwater, (3) glacial ice, and (4) meltwater. Bacteria were the most abundant microbial domain, ranging from 0.2–1.4 × 10^7^ 16S rRNA gene copies mL^-1^ in pond/lake and meltwater, 0.1-7.8 × 10^6^ copies mL^-1^ in groundwater and less than 10^4^ copies mL^-1^ in ice. The number of archaeal 16S and fungal 5.8S rRNA genes was generally less than 6.0 × 10^3^ and 1.5 × 10^3^, respectively. N_2_-fixing and methane-oxidizing Actinomycetes, Bacteroidetes and Verrucomicrobia were the dominant microorganisms in the pond/lake samples, whereas iron reducing *Desulfosporosinus* sp. dominated the deep anaerobic groundwater. The glacial ice was inhabited by Cyanobacteria, which were mostly Chloroplast-like. The meltwater contained methano- and methylotrophic Proteobacteria, but had also high relative abundances of the nano-sized Parcubacteria. The archaea composed approximately 1% of the 16S rRNA gene pool in the pond/lake samples with nano-sized Woesearchaeota as the dominating taxon, while in the other sample types archaea were almost negligent. Fungi were also most common in the pond/lake communities, were zoospore-forming Chytridiomycetes dominated. Our results show highly diverse microbial communities inhabiting the different cold Greenlandic aqueous environments and show clear segregation of the microbial communities according to habitat, with distinctive dominating metabolic features specifically inhabiting defined environmental niches and a high relative abundance of putatively parasitic or symbiotic nano-sized taxa.

## Introduction

Arctic environments are highly susceptible to the effects of global climate change. The effects can be seen as increased glacier melt and glacier thinning, melting of the permafrost to greater depths and increased generation of melt water (Hanna et al., 2008). The permafrost has a major impact on hydrology in Arctic regions since it separates the surface water from the deep subsurface water, providing to two from each other separate water environments (e.g., White et al., 2007; Bosson et al., 2013; Kane et al., 2013). However, in specific spots or areas called taliks, flow between surface and deep subsurface water may be possible because the ground remains unfrozen below these taliks throughout the year.

Warming of the arctic has a profound effect on arctic lake ecosystems. Even a few degrees raise in temperature may increase the open water period of arctic lakes, which may result in greater primary production rates (Vincent et al., 2009). Greater primary production is followed by increase (blooms) in biota consuming the primary produced compounds, which affect the light penetration to deeper water layers and eventually increases the amount of dead biomass sinking to the bottom of the lake where it is decomposed and causes alterations in the oxygen levels of the lake (Scheffer and Carpenter, 2003). Decreasing light penetration would also lead to decrease in the original rate of primary production, turning the lakes from CO_2_ sinks to CO_2_ sources (Mariash et al., 2018). Increased organic matter could severely change the food web composition in oligotrophic lakes from mostly bacteria driven in oligotrophic water to microbial eukaryotic driven with increasing organic carbon content (Cotner and Biddanda, 2002; Mariash et al., 2018). Heterotrophic bacteria have been shown to dominate the microbial communities in oligotrophic aquatic environments, such as arctic lakes, where they have crucial roles in the mineralization of primary produced organic compounds as well as recycling of carbon (reviewed by Cotner and Biddanda, 2002). In oligotrophic lakes the organic carbon is generally dissolved, in comparison to eutrophic lakes, where the organic carbon is bound in particulate matter (Biddanda et al., 2001).

Bioavailable nitrogen is introduced to the ecosystem through nitrogen fixation. In Svalbard tundra and lake sediment the availability of ammonia, nitrate, organic carbon and organic nitrogen were strong drivers for the diversity of the bacterial communities (Wang et al., 2016). In Greenlandic tundra soil, moisture was the main factor driving the nitrogen fixation, but increasing soil temperature had a negative effect on the nitrogen fixation rate (Rousk et al., 2018). Nevertheless, bedrock may also be a significant source of nitrogen compounds in aquatic ecosystems as different types of rock, e.g., metasedimentary rock types, may contain significant (up to 1 g kg^-1^) concentrations of nitrogen (Holloway and Dahlgren, 2002). In tundra ponds in Alaska, phosphorus was the more critically limiting nutrient, whereas nitrogen was constantly supplied from the bottom sediment (Alexander et al., 1989). Severe phosphorus limitation was also reported for two lakes in SW Greenland, especially in spring (Brutemark et al., 2006). A study on more than 50 lakes and other surface water types (supraglacial and subglacial meltwater) in the Kangarlussuaq area also reported severe P and N limitations in the water (Henkemans, 2016). Thus, oligotrophic lakes are highly susceptible to increased input of nitrogen and phosphorus, which may lead to increased growth of the algal communities and this may lead to an elevation in the nitrogen and phosphorus concentrations in the water resulting from decaying biomass (Ogbebo et al., 2009).

The motivation for studying the microbial communities in aquatic environments above and below the permafrost in Greenland is connected to the safety of deep geological repositories (DGRs) for high-level radioactive waste. DGRs are designed to keep the spent nuclear fuel isolated from the surface biosphere on a time scale of hundreds of thousands of years. Over this time frame, glacial conditions are expected in northern latitudes, such as the Nordic countries and Canada (e.g.,SKB, 2011; Posiva, 2012). A thick ice cover may influence groundwater flow due to increased ice load. Melt water from the ice may also penetrate in to the bedrock environment bringing nutrients and oxygen down to repository depths (e.g., SKB, 2011; Posiva, 2012). Greenland with its ice sheet and permafrost was used as an analogous environment in the Greenland Analog Project (GAP) to investigate the hydrological, hydrogeological and geochemical processes that a nordic DGR can experience during glacial conditions resembling the retreat phase of a glacial cycle (Claesson Liljedahl et al., 2016; Harper et al., 2016). The aims were to investigate how and if the surface waters influence the deep groundwater using both geochemical and microbiological tools. In addition, we aimed to characterize the composition of arctic microbial communities in different aquatic environments and estimate the metabolic potential of these communities in order to gain more information on the little studied arctic microbial flora. Our hypotheses were; (1) the deep subsurface microbial communities are distinctly different from the surface water communities, and (2) the different environment types contain defined, environment-specific communities, even though the environments may be in direct contact with each other, such as the inflow to the Talik lake and the Talik lake water.

## Materials and Methods

### Site Description

**FIGURE 1:**
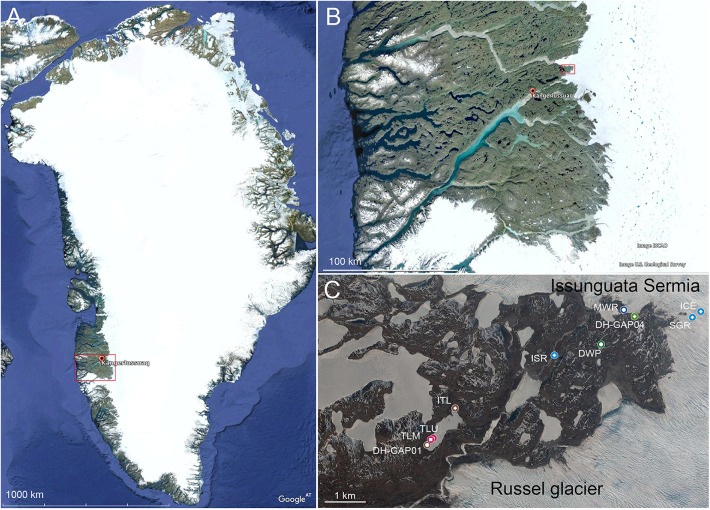
The study area is located on the west coast of Greenland **(A)** in the Kangarlussuaq area **(B)**, which is indicated by the red square in **A**. The sampling area **(C)** is indicated by a red square in **B**. The sampling sites are indicated by colored symbols in **C**. The sample codes are explained in Table 1. The white bar in the lower left corner of the figures represent 1000, 100, and 1 km in **A**, **B**, and **C**, respectively. DWP, drill water pond; TLU, talik lake up; TLM, talik lake mid; DH-GAP01, drillhole DH-GAP01; DH-GAP04, drillhole DH-GAP04; ICE, glacial ice from Issunguata Sermia; SGR, Supra Glacial River; MWR, melt water river directly below the glacier; ISR, Issunguata Sermia River; ITL, inflow to Talik lake. Maps from Google Earth 2017.

The Kangerlussuaq area is situated on the west coast of Greenland, approximately 50 km north of the Arctic circle. Local bedrock belongs to the Nagssugtoqidian Orogen area, which is composed of 1800–1900 Ma old Archaean ortho-gneisses containing minor amounts of amphibolite and metasedimentary rocks, that have undergone metamorphosis during the Palaeo-Proterozoic (van Gool et al., 2002; Garde and Marker, 2010). The nearest ice tongues of the Greenland Ice Sheet (GrIS) are situated only 20 km east of the Kangerlussuaq village (Figure 1). Here the ice sheet terminates on land at a distance of 150 km from the coast. The mean annual air temperature is -5.1°C and the annual precipitation 173 mm for the period 1977–2011 (Cappelen, 2018). The two main outlet glaciers in the area are Isunnguata Sermia and the Russell glacier (Figure 1). In the GAP study area, the ice sheet thickness reaches approximately 1500 m with a mean value of approximately 800 m (Lindbäck et al., 2014). The ice flow is generally in the direction from east to west with a mean surface velocity of the 150 m/year. The outlet glaciers here are land-terminated and isolated from marine influence.

The vegetation consists of dwarf-shrub heath, which grow lower and less dense with increasing altitude and proximity to the ice sheet. The ice sheet melt season, with surface melt and runoff, lasts from May to September, during which time the ice sheet loses 3–4 m of thickness. The climate in the study area in low Arctic continental with continuous permafrost and is highly impacted by the presence of the GrIS. The study area is described in detail in Claesson Liljedahl et al. (2016) and Harper et al. (2016).

Surface waters (glacial ice and meltwaters, melt water ponds, lakes, rivers) and subsurface waters (deep groundwater) have been extensively studied in the Kangarlussuaq area (Henkemans, 2016). The waters are typically highly nitrogen deficient with nitrate concentrations generally below the detection limit of 0.2 mg L^-1^. The surface waters are furthermore severely depleted in phosphorus, with P concentrations generally below 10 μg L^-1^. However, the deep groundwater from three different sections (described below) of borehole and DH-GAP04 was shown to contain 42–4320 μg L^-1^ P, although the concentration of P in groundwater from borehole DH-GAP01 was below 10 μg L^-1^.

A total of 12 different samples, representing lake and pond water (samples DWP, TLU, TLM), deep subsurface water (samples DH-GAP01, DH-GAP04.up/.mid/.low) and ice and meltwater (samples ICE, SGR, MWR, ISR, and ITL) were collected during September 6–14, 2014 (Table 1 and Figure 1) when the permafrost is thinnest. The Talik lake area, from where samples ITL, TLU, TLM, and DH-GAP01 originate is situated at an elevation of 369 m above sea level (masl) measured from the lake surface, and approximately 800 m from the ice sheet margin. The lake has a surface area of 0.37 km^2^ and a catchment area of 1.56 km^2^ and a maximum depth of 29.9 m. The catchment area is dominated by glacial till and glaciofluvial deposits overlain by eolian silt and fine sand. Water flows into the lake from the melting active layer of the permafrost and may also be influenced by the under lying groundwater (Johansson et al., 2015).

**Table 1:** Sampling sites, dates and coordinates of the study and explanations to the abbreviations (Code) in Figure 1.

Code	Name	Description	Sampling date	Coordinates (UTM 22 WGS84)
DWP	Drill water pond	Pond used as water source for DH-drilling GAP04. Sample from 0–20 cm depth.	8.9.2014	N 7448270 E 0539892 z 427 m
TLU	Talik lake Upper layer	Talik Lake by DH-GAP01, 0–20 cm	11.9.2014	N7445789, E0535645, z 384 m
TLM	Talik Lake Middle layer	Talik Lake by DH-GAP01, 10 m depth	11.9.2014	N 7445751, E 0535581, z 334 m
DH-GAP01	Drillhole #01 of the Greenland Analog Project	DH-GAP01 borehole, borehole section 129–191 m	6.9.2014	N 7445607, E 0535489
DH-GAP04.up	Drillhole #04 of the Greenland Analog Project upper sample	DH-GAP04 borehole	12.9.2014	N 7449004, E 0540732
DH-GAP04.mid	Drillhole #04 of the Greenland Analog Project middle sample	DH-GAP04 borehole	13.9.2014	N 7449004, E 0540732
DH-GAP04.low	Drillhole #04 of the Greenland Analog Project lower sample	DH-GAP04 borehole	14.9.2014	N 7449004, E 0540732
ICE	Glacial ice	Isunnguata Sermia	10.9.2014	N 7449156, E 0542424, z 517 m
SGR	Supra glacial river	Isunnguata Sermia	10.9.2014	N 7449007, E 0542209, z 489 m
MWR	Melt water river	Discharge directly below Isunnguata Sermia glacier at DH-GAP04	9.9.2014	N 7449170, E 0540460, z 403 m
ISR	Isunnguata Sermia river	Downriver after meltwater pond	14.9.2014	N 7447968, E 0538703
ITL	Inflow to Talik lake	Infiltration of permafrost active layer meltwater into Talik Lake	12.9.2014	N 7446570, E 0536194, z 384 m


Drill hole DH-GAP01 was drilled in 2009. It is situated 20 m from the lake and was drilled at an angle to reach below the lake. The drill hole has a length of 221 m and reaches a vertical depth of 191 m below land surface. It was drilled using sodium fluorescein (C_2_OH_10_Na_2_O_5_) spiked water from the Talik lake. An inflatable packer is installed at 129 m vertical depth below land surface (drill hole length 150 m), allowing for sample collection from 129 – 191 m vertical depth, yielding a sampling section with a volume of 174 L. Sample intake and sensors for *in situ* pressure, temperature and electric conductivity (EC) are located at 161 m borehole length (138 m vertical depth) (Kontula et al., 2016). The lithology of this section consists mostly of felsic gneiss, with inclusions of intermediate gneiss at drill hole length 110–130 m and mafic gneiss below 180 m (Pere, 2014). Fractures are frequent and the fracture filling mineral is pyrite. The groundwater is anaerobic.

Drill hole DH-GAP04 is situated just at the margin of the Isunnguata Sermia outlet glacier (Figure 1). This drill hole was drilled in June 2011, has a length of 687 m and reaches a vertical depth of 645 m below land surface. Water from the Drill Water Pond (DWP), a small pond situated at approximately 1 km southwest of DH-GAP04, spiked with sodium fluorescein, was used as drilling water. Two inflatable packers are installed dividing the drill hole into three sections. The packers are installed at vertical depths of 561 and 571 m below land surface (drill hole lengths 593.5 and 605.5 m, respectively), isolating a 44 L section (DH-GAP04.mid) including two fracture zones (Harper et al., 2016). The section above the upper packer (DH-GAP04.up) extends from the base of the permafrost at 400 m depth to the upper packer, forming a 840 L drill hole section with groundwater inflow from 3 fracture systems (borehole length 548, 551, and 584 m, vertical depth 518, 522, 554 m, respectively), with a sample intake, EC and *in situ* pressure sensors installed at 541 m borehole length (512 m vertical depth). The lower section (DH-GAP04.low) has a volume of approximately 350 L containing 3 weak fractures at 638, 670, and 682 m drill hole length, equal to 604, 632, and 644 m vertical depth. Sample intake, EC and *in situ* pressure sensors are located within 2 m of the lower packer (Kontula et al., 2016). The lithology of the DH-GAP04 drill hole was intermediate gneiss at the sampling depths (Pere, 2014). The fracture filling mineral is gypsum at the depth of sampling. For more details on boreholes DH-GAP01 and DH-GAP04, see e.g., Pere (2014), Harper et al. (2016), Kontula et al. (2016). The groundwater is anaerobic.

### Sampling for Chemistry

The temperature, pH and conductivity of the water samples were measured immediately in the field with a portable pH, conductivity and temperature meter (Oakton), with exception of the ice sample, which was thawed in the laboratory before measurement. Sodium fluorescein had been added to the drilling fluid when the drill holes were drilled in order to monitor the possible contamination of the deep groundwater by the drilling fluid. By purging the drill holes and allowing them to fill with groundwater from the fracture zones, the sodium fluorescein concentration and thus the drilling fluid impact eventually decreases. Samples for measuring the fluorescein content were collected into plastic bottles and wrapped in aluminum foil in order to protect the fluorescein from deterioration. Water samples for analysis of anions were collected as such directly in to unused factory-clean plastic bottles (Nalgene) and were filtered in the laboratory prior to analysis through 0.45 μm pore-size cellulose acetate filters. Samples for cationic analyses were filtered in the field through 0.45 μm pore-size cellulose acetate filters in to factory-clean plastic bottles (Nalgene) and acidified with ultra-clean nitric acid that had been rinsed three times with filtered sample water before sample collection. The anaerobic samples from DH-GAP01 and DH-GAP04 samples were collected anaerobically under nitrogen atmosphere. The samples for geochemical analyses were sent to Teollisuuden Voima Oyj (TVO), Finland, where the cations were analyzed with ICP-OES (iCAP 6500 Thermo Fisher Scientific). Anions (Br, Cl, F, NO_3_, and SO_4_) were analyzed with ion chromatography (DIONEX ICS-200). The alkalinity of the samples was determined with titrimetric analysis (Metrohm 905 Titrando) and the concentration of HCO_3_ was calculated from this analysis. Non-purgeable organic carbon (NPOC) and Dissolved inorganic carbon (DIC) were filtered through 0.45 μm pore-size cellulose acetate filters and analyzed with a TOC analyzer at TVO with Shimadzu TOC-V CPH. Chemical analyses were done from three subsamples of each sample.

### Samples for Microbiology

Water samples were collected in triplicate from each sampling site. The biomass from the water samples was collected on to Sterivex^TM^ filter units using a peristaltic pump equipped with sterile silicone tubing. The samples on the filters were immediately fixed with 5 mL MoBio LifeGuard^TM^ solution in order to preserve the nucleic acids and placed on coolers in a cooling box for a maximum time of 6 h until the samples could be placed in a freezer at -20°C. The samples were transported frozen from Greenland to Finland for processing.

In addition, two parallel 50 mL water samples were collected from each site into sterile, oxygen-free glass infusion bottles equipped with an air tight butyl rubber stopper (Bellco) and aluminum crimp cap. The water samples were collected in sterile syringes equipped with a hypodermic needle. The water samples were inserted into the sealed bottle by pushing the needle through the rubber stopper. These live water samples were kept refrigerated at 4°C and transported on ice to Finland. The storage time before processing, due to logistical necessity, was at most 2 weeks.

### Estimation of Number of Cultivable Microorganisms

Nutrient agar, TSA and R2A (Sigma) agar plates for cultivation of heterotrophic microorganisms were prepared from ready media mixes according to the manufacturer’s recommendations. In addition, autotrophic microorganisms were targeted using modified Medium 72 agar plates^1^ without methanol. A ten-fold dilution series was prepared from each sample in sterile 0.9% NaCl solution. Aliquots of 100 μl of the undiluted sample, 10^-1^ and 10^-2^ dilutions were spread on duplicate agar plates in a laminar flow hood. The agar plates were incubated at +1°C (+/-1°C) and the appearance of colonies was checked for weekly.

### Biolog AM

The capacity of the indigenous microbial flora of the different water samples for hydrolyzing carbon and nitrogen compounds was tested on Biolog AM 96-well plates (Biolog, Hayward, CA, United States) intended also for anaerobic microorganisms. The Biolog AM plates were prepared in an anaerobic cabinet in order to protect the plates and the samples from oxygen. The sealed sample bottles were opened in an anaerobic cabinet and aliquots 100 μl sample water were pipetted into each well on four parallel Biolog AM plates per sample. Two of the four plates were inserted into anaerobic pouches equipped with an oxygen indicator in order to maintain anoxic conditions during incubation, and two of the plates were incubated in ambient atmosphere. The Biolog AM plates were kept at 10°C and checked weekly for changes in color in the wells.

### Nucleic Acid Isolation

For DNA extraction the Sterivex filter units were opened in a laminar flow hood using sterilized pliers. The membranes were removed with sterile scalpels and tweezers and put in 15 mL sterile screw cap cone tubes (Corning) for DNA extraction. DNA was extracted from the filters using the NucleoSpin Soil DNA isolation kit (Macherey-Nagel, Germany). First the beads from one bead tube were inserted to the cone tube containing the Sterivex^TM^ membrane and two reaction volumes of SL1 buffer was added to the tube, and the sealed tubes were horizontally shaken using a vortex shaker for 10 min. After 5 min centrifugation at 4000 rpm in an Eppendorf 5810R table top centrifuge the supernatant was collected and the extraction procedure continued as recommended by the manufacturer. The DNA was eluted in 50 μl of buffer SE and the DNA concentrations were measured using a Nanodrop 1000 spectrophotometer (Thermo Fisher Scientific). The mean DNA concentrations for the different sample types varied between 4.0 ± 1.0 ng μl^-1^ in DH-GAP04-UP to 10.2 ± 6.8 ng μl^-1^ in ITL. The extracted DNA was stored at -80°C.

### Estimation of Microbial Community Size by Quantitative PCR

The size of the microbial community in the different sampling sites was estimated by bacterial and archaeal 16S rRNA gene qPCR as described in Bomberg et al. (2016). The fungal community sizes were estimated targeting the fungal 5.8 rRNA gene using a TaqMan approach, using primers 5.8F1 and 5.8R1 and probe 5.8P1 (Haugland and Vesper, 2002) as described in Rajala et al. (2016).

### Amplicon Library Preparation

The amplification libraries for high throughput sequencing with Ion Torrent PGM were prepared by PCR from the DNA samples. Bacterial 16S genes were amplified with primers S-D-Bact-0341-b-S-17/S-D-Bact-0785-a-A-21 (Herlemann et al., 2011), targeting the variable region V3-V4 region of the 16S rDNA gene, archaeal 16S genes with primers S-D-Arch-0349-a-S-17/S-D-Arch-0787-a-A-20 (Klindworth et al., 2013), targeting the V4 region of the gene and fungal internal transcribed spacer (ITS) gene markers with primer pair ITS1 and ITS2 targeting the fungal ITS1 region (White et al., 1990; Gardes and Bruns, 1993). PCR amplification was performed in parallel 25 μl reactions for every sample containing 1× MyTaq^TM^ Red Mix (Bioline, London, United Kingdom, 20 pmol of each primer, up to 25 μl molecular-biology-grade water (Sigma) and 2 μl of template. The PCR program consisted of an initial denaturation step at 95°C for 3 min, 35 cycles for bacteria and fungi and 40 cycles for archaea of 15 s at 95°C, 15 s at 50°C, and 15 s at 72°C. A final elongation step of 30 s was performed at 72°C. The PCR products were verified with agarose gel electrophoresis. Amplicons were sent to Ion Torrent sequencing with PGM equipment (Bioser, Oulu, Finland) and amplicons were purified before sequencing by the staff at Bioser. The sequences have been submitted to the European Nucleotide Archive (ENA) under Study Accession Number PRJEB30970.

### Sequence Processing and Analysis

The fastq sequence files obtained from Ion Torrent sequencing were first converted to fasta and qual files using the QIIME v. 1.9 software (Caporaso et al., 2010) and the sequence analysis was continued using the mothur software, v. 1.33.4 (Schloss et al., 2009). Adapters, barcodes and primers were removed and the sequence reads were trimmed to a minimum length of 250 nucleotides using mothur. No barcode differences, no ambiguous nucleotides and a maximum of 8 nucleotide homopolymers were allowed. A qwindowaverage of 25 and a qwindowsize of 50 were used on the PGM read data in order to remove erroneous reads from the data set. Chimeric sequence reads were removed with Chimera Slayer in mothur using the Silva 128 database as template (Quast et al., 2012). A phylip distance matrix was built according to the aligned sequences using the dist.seqs command in mothur with a cutoff value of 0.03. Sequence reads were clustered in to operational taxonomic units (OTU) sharing 97% identity within each OTU. The OTUs were classified against the Silva.seed_v128 database in mothur using the wang classification method. Functional profiles for the bacterial and archaeal communities were predicted using FAPROTAX (Louca et al., 2016).

ITS1 sequences were analyzed using the QIIME v 1.9 software. Adapters, barcodes and primers were removed from the sequence reads, and chimeric sequence reads were removed from the dataset with the USEARCH algorithm (Edgar, 2010) by *de novo* detection and through similarity searches against the UNITE reference dataset (Version sh_refs_qiime_ver7_dynamic_s_20.11.2016_dev) (Kõljalg et al., 2013; Unite Community, 2017; Nilsson et al., 2018) with fungal sequences. Sequences that failed to hit the sequence database with a minimum of 60% sequence identity were discarded as sequencing errors. The open reference strategy in QIIME was used for OTU picking and taxonomic classification of the sequences.

Alpha-diversity measures (number of OTUs detected in the dataset – observed OTUs, estimated number of OTUs that could in total be detected in the environment from which the data set originates – Chao1 OTU richness, and the Shannon diversity index describing the OTU richness and evenness of OTU distribution in the examined environment) were calculated based on the absolute number of sequence reads per OTU using the Phyloseq package in R (McMurdie and Holmes, 2013; R Development Core Team, 2018) and visualized using ggplot2. The similarity of the archaeal, bacterial and fungal communities between the different sample sites was tested by principal coordinate analysis (PCoA) using the Phyloseq package in R using the Bray-Curtis dissimilarity model. Eigen values for the variance explained by the PCoA dimensions were calculated on 999 random repeats.

Significant difference between the mean number of bacterial and archaeal 16S rRNA gene copies, fungal 5.8S rRNA copies and colony forming units (cfu) per mL sample water was tested using one-way ANOVA, Tukey’s Q test and Kruskal-Wallis test using the PAST3 software (Hammer et al., 2009). In addition, the differences between microbial communities above and below the permafrost and between different sample types was tested with PERMANOVA using the Bray-Curtis dissimilarity model and 9999 permutations in PAST3.

## Results

### Chemical Parameters of the Samples

The pH of the different water samples varied between 6.3 in the ICE sample (measured from molten ice in the laboratory) and 8.7 in the DH-GAP04.mid sample (Table 2). The conductivity (EC) of the water was highest, 3.71 mS/cm, in the DH-GAP04.low sample and lowest in the ICE and melt water samples. The deep subsurface DH-GAP04.up, .mid, and .low samples had the highest salinity, total concentration of sulfur and sulfate, while the pond and lake water contained the highest concentrations of dissolved inorganic carbon (DIC), bicarbonate and non-purgeable organic carbon (NPOC) (Table 2).

**Table 2:** The chemical characteristics of the sampled water.

Sample	unit	DWP	TLU	TLM	DH-GAP01	DH-GAP04.up	DH-GAP04.mid	DH-GAP04.low	ICE	SGR	MWR	ISR	ITL
pH (field)		8.4	8.3	8.4	8.6	8.5	8.7	8.2	6.3^∗^	8.1	7.5	7.4	6.9
Temperature (field)	°C	6.7	7.4	6.4	7.0	11.3	10.5	10.6	–	0.4	0.4	6.8	3.4
Conductivity (field)	mS/cm	0.18	0.19	0.19	0.82	2.68	2.66	3.71	0.00^∗^	0.00	0.02	0.19	0.17
Gran titration, HCl consumption	mmol/L	1.5	1.6	1.6	n.a.	0.21	0.23	0.31	<0.03	<0.03	0.09	0.07	0.41
NPOC	mg/L	16	7.0	7.0	n.a.	4.6	4.0	0.8	6.9	<0.3	0.5	0.7	21
DIC	mg/L	16	17	17	n.a.	2.2	2.3	3.2	0.3	<0.3	1.1	0.9	4.8
Charge balance DIC	%	7.06	7.02	7.20	3.73	-0.39	-1.15	-1.14	-10.02	100.00	12.53	11.74	6.48
Charge balance HCO_3_	%	2.17	1.86	2.02	3.73	-0.44	-1.21	-1.19	1.90	-31.11	13.04	13.44	6.12
Nitrogen total	mg/L	1.1	0.49	0.50	n.a.	0.50	0.27	0.053	0.042	0.052	0.30	0.34	1.1
Nitrate	mg/L	<0.4	<0.4	<0.4	<0.4	<0.4	<0.4	<0.4	<0.4	<0.4	<0.4	<0.4	<0.4
Iron	mg/L	0.11	0.009	0.020	0.027	0.024	0.018	0.056	0.064	0.017	0.15	0.15	0.24
Sulfur total	mg/L	2.1	1.3	1.3	120	460	440	660	<0.3	<0.3	0.72	0.79	14
Sulfate	mg/L	6.0	3.5	3.5	370	1410	1370	1990	<0.1	<0.1	2.1	2.3	41
TDS	mg/L	144	148	148	568	2195	2147	3123	2	1	13	12	108
Aluminum	μg/L	6	2	8	10	27	9	3	26	14	150	150	97
Bicarbonate	mg/L	92	98	98	n.a.	13	14	19	1.2	0.6	5.5	4.3	25
Bromid, Br	mg/L	<0.1	<0.1	<0.1	0.2	1.2	1.3	2.1	<0.1	<0.1	0.3	<0.1	<0.1
Fluorid, F	mg/L	0.2	0.3	0.3	0.6	0.2	0.2	0.1	<0.1	<0.1	<0.1	<0.1	0.1
Potassium	mg/L	5.2	6.2	6.2	2.0	4.1	6.7	5.5	<0.1	<0.1	0.4	0.4	1.4
Calsium	mg/L	14	15	15	99	370	330	430	<0.2	<0.2	1.9	1.7	14
Chloride,	mg/L	8.5	8.6	8.5	7.7	100	110	170	<0.2	<0.2	<0.2	0.3	4.6
Magnesium	mg/L	10	9.9	9.9	3.3	25	31	60	0.01	0.03	0.38	0.40	7.5
Sodium,	mg/L	7.0	6.6	6.6	74	260	270	430	0.3	<0.2	0.4	0.3	4.7
SiO_2_	mg/L	0.70	0.45	0.46	8.1	5.4	6.4	7.9	0.09	0.29	1.9	1.6	8.8
Strontium	mg/L	0.05	0.05	0.05	2.6	6.5	6.3	8.2	0.01	0.01	0.02	0.02	0.05
Sodium fluorescein	mg/L	n.d.	n.d.	n.d.	<1	76	67	2.6	n.d.	n.d.	n.d.	n.d.	n.d.


*^∗^ measured in the laboratory, n.a., not available; n.d., not determined.*

### Microbial Biomass

**FIGURE 2:**
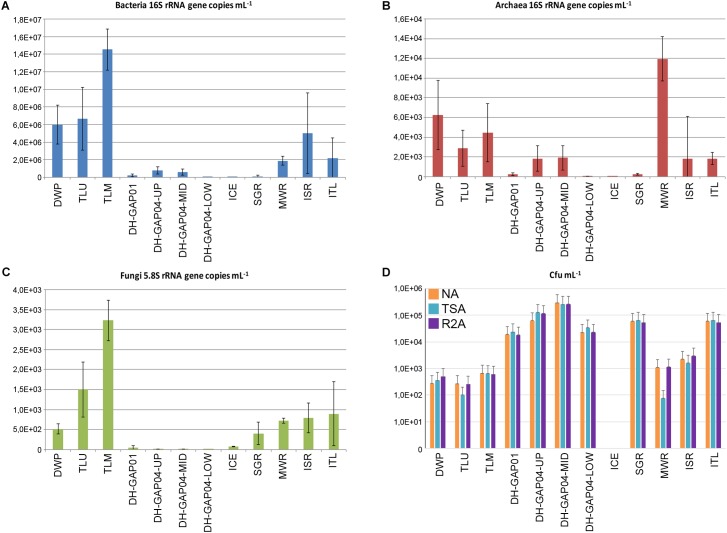
The mean abundance of **(A)** bacteria, **(B)** archaea, **(C)** fungi estimated by quantifying the bacterial and archaeal 16S rRNA gene copies and the fungal 5.8S rRNA gene copies mL^-1^ sample water. **(D)** The mean number of colony forming units (cfu) mL^-1^ in the different water samples grown on Nutrient Agar (NA), Tryptic Soy AgR (TSA) and R2A agar. The error bars show standard deviation. In **(A–C)** the number of biological replicants was 3, of which 3 individual qPCR reactions were performed for each replicant. In **(D)** the mean number of cfu is based on dilution series including 4–6 plates per sample.

The number of bacterial and archaeal 16S rRNA gene copies, fungal 5.8S rRNA gene copies and number of colony forming units (cfu) varied greatly between the different sample types (Figure 2). The concentration of bacterial 16S rRNA genes varied between 1.5 × 10^7^ mL^-1^ in TLM and 1.4 × 10^4^ mL^-1^ in DH-GAP04.low, while the concentration of archaeal 16S rRNA genes was highest in MWR, 1.2 × 10^4^ and lowest in ICE, 1.5 × 10^1^ mL^-1^. The fungal 5.8S rRNA gene concentration was highest, 3.2 × 10^3^ in TLM and lowest, 5.0 mL^-1^, in DH-GAP04.low. The amount of culturable microorganisms varied on NA between 2.7 × 10^1^ in TLM and 2.9 × 10^5^ cfu mL^-1^ in DH-GAP04.mid, on TSA between 7.5 × 10^1^ in MWR and 2.6 × 10^5^ cfu mL^-1^ in DH-GAP04.mid, and on R2A between 2.6 × 10^2^ in TLU and 2.6 × 10^5^ cfu mL^-1^ in DH-GAP04.mid. No microbial colonies were obtained from the ICE sample. No colonies were detected on the modified M72 medium targeting autotrophs.

The highest concentration of bacterial 16S rRNA genes and fungal 5.8S rRNA genes correlated positively and significantly (*r* > 0.8, *p* < 0.02 and *r* > 0.6, *p* < 0.02 for bacteria and fungi, respectively) with the highest concentrations of DIC and bicarbonate. The highest number of cfu:s obtained on TSA correlated positively and significantly (*r* > 0.59, *p* < 0.05) with the highest concentration of Ca, S_tot_, Sr, SO_4_, and TDS, and the highest number of cfu:s on R2A correlated positively and significantly (*r* > 0.6, *p* < 0.04) with the highest concentration of Ca.

### Alpha Diversity

The number of bacterial, archaeal and fungal sequences varied in the samples between 3.0 × 10^3^–7.9 × 10^3^, 1.0 × 10^1^–8.6 × 10^3^ and 3.0 × 10^3^–7.9 × 10^3^, respectively (Supplementary Table S1 and Supplementary Figure S1). The highest number of observed bacterial OTUs (1.5 × 10^3^) was observed in MWR, while the lowest number (2.2–4.5 × 10^2^) were detected in the deep subsurface samples and ITL. Nevertheless, the highest number of Chao1 estimated bacterial OTUs (4.6 × 10^3^) was estimated for the ITL sample and the lowest for the deep subsurface samples. The ITL bacterial community also had the highest Shannon diversity index of 8.8 while the lowest Shannon diversity indices 3.6–6.1) were calculated for the deep subsurface samples and ICE.

The number of archaeal observed OTUs was highest in the DWP, MWR, ISR and ITL (8.8 × 10^2^ – 1.2 × 10^3^) and lowest in the deep subsurface and ICE, where the number of archaeal sequences was also low. The highest number of Chao1 estimated archaeal OTUs was also detected in DWP. The Shannon diversity index was highest in the lake/pond samples and MWR, ISR, and ITL and lowest in the deep subsurface and ICE samples.

The highest number of fungal ITS OTUs was observed in the lake/pond samples and MWR, ISR and ITL, while the lowest OTU numbers were obtained from the deep subsurface samples, ICE and SGR. The same was seen in the Chao1 estimations, with the exception that DH-GAP01 and ICE were also estimated to contain approximately 1.0 × 10^3^ OTUs. The Shannon index for the fungal communities was above 4 in all other samples except TLU and the deep subsurface samples.

### Bacterial Community

**FIGURE 3:**
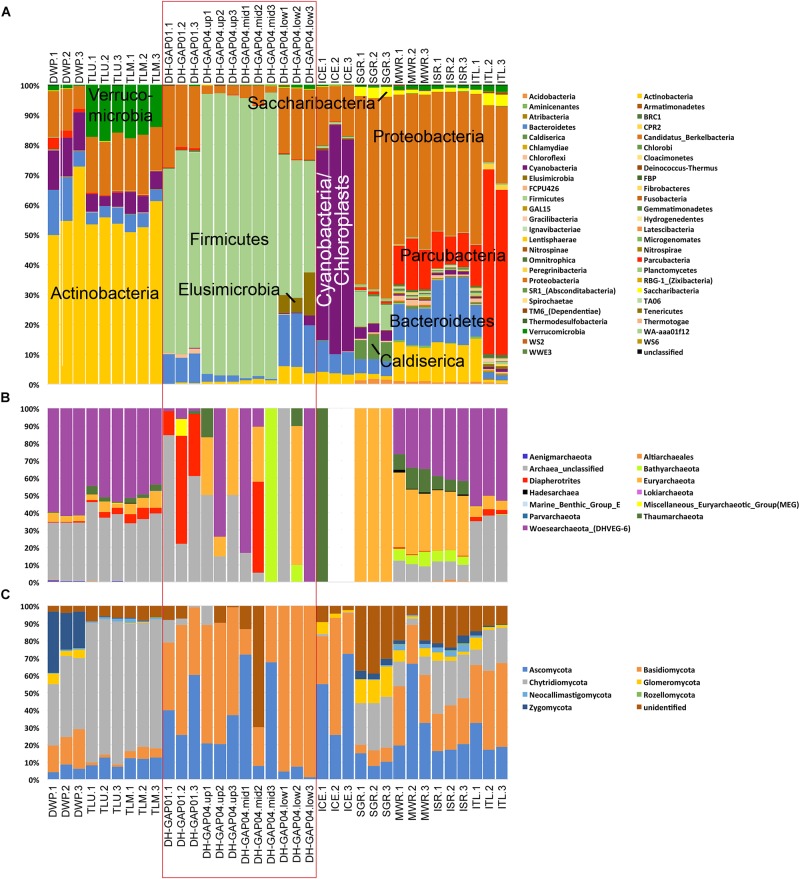
The relative abundance of **(A)** bacterial, **(B)** archaeal, and **(C)** fungal phyla identified in the different samples. Note that each sampling site is represented by three biological replicates. The deep subsurface samples are indicated with a red rectangle, the pond/lake samples are presented to the left and the ice and melt water samples to the right of the rectangle for clarity. The bacteria with the highest relative abundances are indicated in **A**.

The bacterial community profiles differed greatly between the pond/lake, deep subsurface and ice/melt water samples. Altogether 52 bacterial Phyla were detected (Figure 3A). Nevertheless, specific bacterial phyla clearly dominated in the different habitats. In the pond/lake samples (DWP, TLU, and TLM), the majority of the bacterial communities consisted of Actinobacteria (Figure 3A), especially taxa belonging to the family Sporichthyaceae (unidentified Sporichthyaceae 15–26% and hgcl clade 18 – 28% of the bacterial 16S rRNA gene sequence reads, Supplementary Figure S2A), and Candidate genus *Planktophila* (4.8 – 8.2%). In addition, the Talik lake samples (TLU and TLM) contained a large proportion of Verrucomicrobia sequences belonging to the genus *Methylacidiphilum* (11–12%) (Supplementary Figure S2B). Alphaproteobacteria belonging to the SAR11 clade were present as a major group in the Talik lake (9.4 – 11.4%) and as a small fraction (1.4%) also in the DWP sample.

In the deep subsurface samples, the major bacterial constituent was Firmicutes belonging to the genus *Desulfosporosinus* (23 – 61% of the sequence reads, Supplementary Figure S2C), and the DH-GAP04 samples also contained Thermoanaerobacterales bacteria classified as family SRB2 (13 – 37% of the sequence reads). Nevertheless, DH-GAP01 also contained 3% deltaproteobacteria belonging to the *Desulfovibrio*, 5.1 and 7.2% alphaproteobacteria belonging to *Janthinobacteria* and *Undibacteria*, respectively (Supplementary Figure S3A).

The bacterial community of the ICE consisted mostly of Cyanobacteria (63.5 – 76.6%) (Figure 3A) and proteobacteria belonging to the *Polaromnas* (10%) (Supplementary Figure S3B).

Proteobacteria were the most abundant bacteria in SGR, MWR and ISR (46 – 67%, Supplementary Figure S3B). Betaproteobacteria of the genera *Albiferax* and *Polaromonas* were detected in all three sample types (4.8 – 9% and 1.1–7.4%, respectively). However, SGR had a large proportion of *Massilia* (12.7%) and *Undibacterium* (20.6%), while MWR and ISR had *Rhodoferax* (1.2 – 4.2%) and *Methanotenera* 6.3 – 8.8%). In addition, MWR and ISR had between 10.7 – 17% *Methylobacter* sequences. Actinobacteria belonging to the Candidate genus *Planktophila* were present at a relative abundance of 6.1 – 7.1% in these two samples.

In the ITL sample, the majority of the bacterial sequences belonged to Parcubacteria (13.5 – 62% of all bacterial sequence reads) (Figure 3A), of which approximately 15% (of all bacterial sequence reads) belonged to a genus of the *Candidatus* class Nomurabacteria and an 10% to an unidentified Parcubacteria genus. Proteobacteria (19 – 50% of the sequence reads) belonged mostly to Methylobacter (6.4%) (Supplementary Figure S3D).

Bacteroidetes bacteria were present in all samples to some extent, 0.6 – 22%, most of which were Flavobacteria (Figure 3 and Supplementary Figure S2D).

**FIGURE 4:**
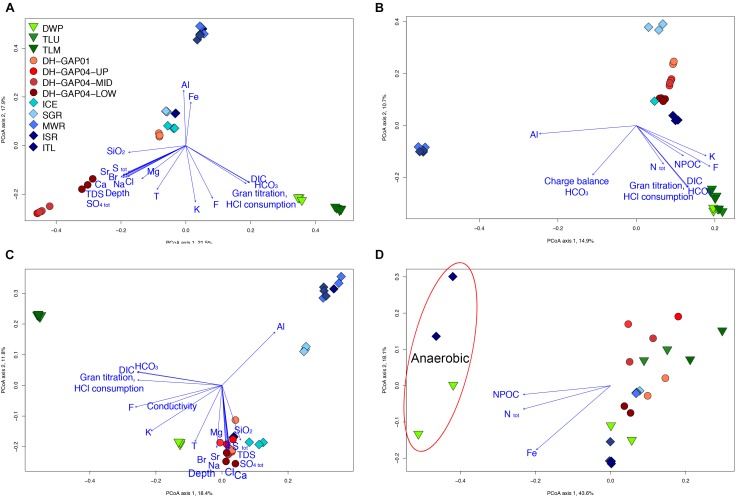
Principal coordinates analysis plots of the **(A)** bacterial, **(B)** archaeal, **(C)** fungal communities, and **(D)** the hydrolysis patterns of substrates in the Biolog AN tests. The key to the samples in all figures is found in **A**. In **D**, the anaerobic Biolog AN tests are indicated with a red oval in the upper left quadrant. Only physicochemical parameters with *p* < 0.0001 **(A–C)** and *p* < 0.001 **(D)** were plotted.

The difference in the bacterial communities could also be seen in the PCoA plots (Figure 4A), where the lake/pond samples fall to the lower right corner of the plot and the DH-GAP04 samples to the lower left. The bacterial community of the lake/pond samples are most strongly affected by the concentration of inorganic carbon (DIC, HCO_3_) and buffering capacity (HCl consumption), while the deep subsurface bacterial communities are affected by salinity, sulfate and total Sulfur concentrations. Most of the ITL, MWR, and ISR cluster to the top of the plot, in the same direction as the concentration of Fe and Al, and the ICE and SGR samples to the middle. Interestingly, the DH-GAP01 samples also fall to the middle of this plot.

The two-way PERMANOVA analysis showed that the bacterial communities of the deep subsurface samples differed significantly (*p* = 0.0001) from the bacterial communities in water samples from above the permafrost. The bacterial communities identified from each sample type, i.e., lake/pond, deep subsurface, and ice and meltwater, also differed significantly (*p* = 0.0001) from each other.

### Archaeal Diversity

Archaea were numerous only in the surface samples, but not in the DH-GAP01 or DH-GAP04 samples, or in ICE (Figure 2B). Altogether 13 archaeal phyla were detected, of which Woesearchaeota showed the highest relative abundance (Figure 3B and Supplementary Figure 4A). Although the archaea were not abundant in the deep drillhole samples, the most common archaea in DH-GAP01 was Ianiarchaeum (37.3%). The DH-GAP04 samples contained Woesearchaeota (24.7 – 33.3%) and *Methanoperedens* (3.3 – 31.5%) (Supplementary Figure S4B). In addition, DH-GAP04.mid had Nanoarchaeum (17.5%) and Bathyarchaeota (33.3%), and DH-GAP04.low had 16.7% *Methanoregula*. The archaeal community of the SGR the archaeal community consisted solely of Euryarchaeota, of which *Methanosaeta* contributed with 37.4% and *Methanospherula* with 58.7%. MWR and ISR contained a rich archaeal community, consisting of the euryarchaeotes *Methanoregula* (17.5 – 19.1%), *Methanoperedens* (5.2 – 7.3%), *Methanosaeta* (3.3 – 5.1%) and TMEG (3.1 – 3.5%), and also Woesearchaeota (31.7 – 40.6%), Thaumarchaeota (6.7 – 13.3%) and Bathyarchaeota (4.8 – 8.5%). The ITL archaeal community was similar to that of the pond/lake communities, containing 53.3% Bathyarchaeota. In all samples, except ICE and SGR, between 10 – 56% of the sequence reads belonged to unidentified Archaea. No archaeal sequences were obtained from the ICE sample.

The archaeal communities divide in to four distinct groups in the PCoA analysis (Figure 4B). The lake/pond samples form a cluster to the lower right corner of the plot, together with the highest concentration of inorganic and organic carbon (NPOC), total N concentration and buffering capacity of the water, while the MWR and ISR cluster together to the left of the plot. The SGR archaeal community cluster to the upper right of the plot while the deep subsurface samples fall in to a loose group to the middle right of the graph. The ITL samples appear to fall closer to the deep subsurface samples, but this is most likely a bias due to the low number of archaeal sequences obtained from the deep subsurface samples.

The two-way PERMANOVA analysis showed that the archaeal identified from samples above and below the permafrost, as well as between different sample types (lake/pond, deep subsurface, and ice and meltwater) differed significantly (*p* = 0.0001).

### Fungal Diversity

The fungal communities consisted of up to eight different fungal phyla. In the pond/lake samples, the fungal communities were dominated with Chytridiomycota (35.5 – 82.4% of all fungal ITS sequence reads in these samples) (Figure 3C), most of which (23.0 – 45.5% of the total number of fungal ITS reads) belonged to unidentified Chytridiomycota (Supplementary Figure S5A). In addition, the chytridiomycotal communities consisted of Powellomyces in all pond/lake samples (9.7 – 19.5%), and Spizellomyces (13.6 – 14.5%) in the Talik lake samples. DWP had also Zygomycota belonging to the *Ramicandelaber* (25.3%) and Basidiomycota (15.4 – 22.7%) belonging to different genera of the class Tremellomycetes (11.7%) (Supplementary Figure S5C). Neocallimastigomycota were represented by Orphinomyces in the lake/pond samples and by Anaeromyces in the metl water samples (Supplementary Figure S5D). The deep subsurface samples, as well as the ICE, contained mostly Ascomycota (20.2 – 72.5%) and Basidiomycota (22.3 – 69.9%), with the exception of the DH-GAP04.low samples, where Ascomycetes were only 1.4 – 7.2%, but Basidiomycetes contributed with 92.6 – 98.6%. In the deep subsurface samples (except DH-GAP04.low) *Cladosporium* was present in all samples (5.9 – 23.4%) (Supplementary Figure S6A). In addition, DH-GAP01 contained a number of Ascomycetes genera, such as an uncultured Helotiales (9.2%), *Phaeosphaeria* (6.2%), *Phaeococcomyces* (4.9%), and *Comoclathris* (4.7%), DH-GAP04.mid contained unidentified Herpotrichiellaceae (41.3%) and the ICE sample *Aspergillus* (4.4%) and unidentified Dothideomycetes (5.0%), in addition to numerous genera present at lower relative abundances (Supplementary Figures S6A–D). In contrast, Basidiomycetes belonging to the *Cryptococcus* were present at relative abundances of 5.6 to 22.3% in the ICE and all deep subsurface samples, except in DH-GAP04.mid, while the genus *Naganisha* was present in the DH-GAP04 and the ICE samples at relative abundances between 6.4 and 30.0, but at only 1.1% in the DH-GAP1 sample (Supplementary Figure S7). In addition, DH-GAP01 contained 7.9% *Collybia* and 17.4% *Mrakiella* and DH-GAP04.up *Hydnum* (5.2%), *Glaciozyma* (14.7%) and all deep subsurface samples and the ICE sample contained 1.5 – 5.3% *Malassezia* sequences (Supplementary Figures S7A–D).

SGR, MWR, ISR, and ITL had the highest diversity of fungal phyla and contained Ascomycota (7.7 – 66.7%), Basidiomycota (5.0 – 48.2%), Chytridiomycota (4.0 – 30.4%), Glomeromycota (0.9 – 17.5%) and a great deal on sequences of unidentified fungi (Supplementary Figure S3C). The main Ascomycetes groups present in all these samples belonged to Cladosporium (2.2 – 4.7%) and unidentified Helotiales (1.0 – 8.5%) (Supplementary Figures S6A,C). The Basidiomycetes in these samples consisted of *Rhodotorula* (1.8 – 4.8%) and unidentified Microbotrymycetes (1.6 – 3.3%), with the exception of SGR (Supplementary Figure S7B). In addition, ITL contained a high relative abundance of *Cronartium* (16.5%) (Supplementary Figure S7B). All the melt water samples, especially SGR, contained a high relative abundance of sequences affiliating with groups of the Glomeraceae (2.9 – 14.5%) not present in the other sample types (Supplementary Figure S5B).

The fungal communities followed the trend of the bacterial and archaeal communities in the PCoA analysis (Figure 4C). The TLU and TLM samples grouped together and the SGR, MWR and ISR formed a cluster of their own. The DH-GAP01, DH-GAP04, ICE and, surprisingly, ITL samples clustered together in the direction of salinity, sulfate and total S concentrations, while the DWP samples fell into a separate group closer to the deep subsurface samples than the other lake samples.

In accordance to the bacterial and archaeal communities, also the fungal communities identified from samples above and below the permafrost, as well as between different sample types (lake/pond, deep subsurface, and ice and meltwater) differed significantly (*p* = 0.0001) from each other when tested with two-way PERMANOVA.

### Metabolic Profiles

**FIGURE 5:**
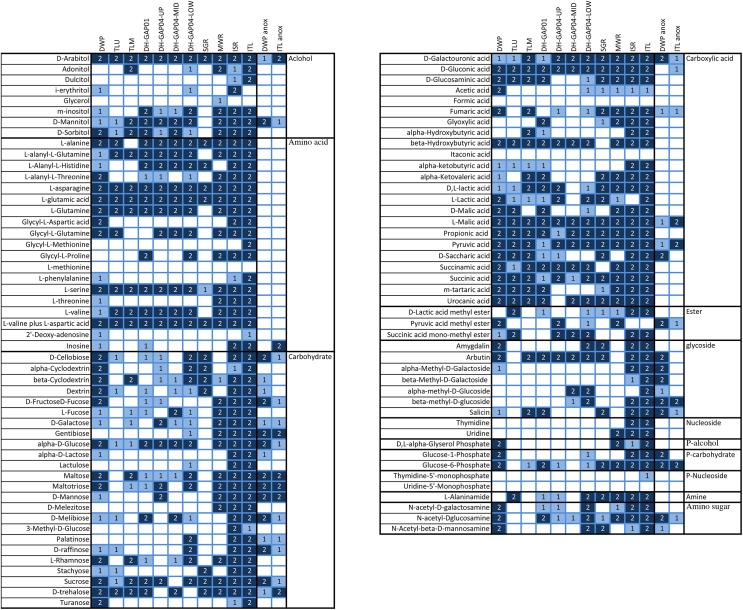
The hydrolysis of substrates on the Biolog AN tests. All tests were done on duplicate test plates. The color coding in the figure indicates whether the hydrolysis occurred on only 1 (light blue) or on both (dark blue) plates. The samples with anaerobic hydrolysis are indicated with anox.

The Biolog AM 96-well substrate plates were used to screen the capacity to utilize different carbon and nitrogen substrates. Substrate hydrolysis was recorded in the oxic tests in all other samples, but ICE (Figure 5). In the anoxic tests, substrates were hydrolyzed only by DWP and ITL communities. Between 32 and 86 of the 95 different substrates were hydrolyzed aerobically (Figure 5). The least number of substrates, 32 and 38, were hydrolyzed by the TLU and DH-GAP04.mid communities, respectively. The highest number of substrates, 80 and 86, were hydrolyzed by the ISR and ITL communities, respectively. In the anaerobic tests, 19 and 28 substrates were hydrolyzed by the ITL and DWP communities, respectively. The two-way PERMANOVA showed that the substrate utilization patterns were distinctly different in the surface water samples (lake/pond, ice and meltwater) compared to the deep subsurface samples (*p* = 0.0009), as well as between the different sample types (lake/pond, ice and meltwater, deep subsurface) (*p* = 0.0001) and the anaerobic vs. aerobic substrate utilization (*p* = 0.0001). In the PCoA analyses the anaerobic DWP and ITL samples clustered together as a separate group from the rest, with the DWP in the direction of the total N, NPOC and Fe concentrations (Figure 4D). The aerobic samples clustered to the lower half of the plot with the samples with the lowest number of hydrolyzed substrates in the left part of the cluster and to the right the samples with the highest number of hydrolyzed substrates. The greatest difference in substrate utilization was observed in the utilization pattern of carbohydrates, as the melt water communities were able to hydrolyze almost all tested substrates, while only D-Cellobiose, beta-Cyclodextrin, Dextrin, alpha-D-Glucose, Maltose, Maltotriose, L-Rhamnose, Sucrose and D-trehalose were hydrolyzed in at least 50% of the reactions from the lake/pond samples, and D-Cellobiose, beta-Cyclodextrin, L-Fructose, D-Galactose, alpha-D-Glucose, Maltose, D-Melibiose, L-Rhamnose, Sucrose and D-trehalose were used in at least 50% of the tests of the deep subsurface samples. Gentibiose was hydrolyzed anaerobically in DWP, but not aerobically. Carboxylic acids were generally hydrolyzed in all sample types, but interestingly, formic acid was not used in any sample. Acetate was used in the DWP, but not in the Talik lake samples. In addition, acetate was sporadically used in the meltwater sample type, but not in the deep subsurface environment, with the exception of one of the replicate reactions from DH-GAP04-LOW. D-Galacturonic acid, D-Glucuronic acid, L-Malic acid, Propionic acid, Pyruvic acid, and Succinic acid were used in all samples, of which D-Galacturonic acid, L-Malic acid and Pyruvic acid were also hydrolyzed anaerobically in DWP and ITL. Beta-hydroxybutyric acid, Succinamic acid, and Urocanic acid was used in all but one sample. Of the alcohols, D-Arabitol was used by all communities, also anaerobically in DWP and ITL. Of the amino acids, L-asparagine, L-glutamic acid, L-serine, and L-valine plus L-aspartic acid were utilized aerobically in all samples, while Glycyl-L-Proline was used only in two of four deep subsurface samples and the MWR, ISR, and ITL. L-Threonine was generally only used in the MWR, ISR, and ITL. Glycosides were sporadically used in all samples, but arbutin was especially popular and used by all, except the TLU and MWR. Interestingly, all tested glycosides, except Amygdalin, was used was used anaerobically in DWP. In contrast, Amygdalin was hydrolyzed aerobically in DWP. Thymidine and Uridine was used only in the melt water samples MWR, ISR and ITL, and only aerobically. DL-alpha-Glyserol Phosphate was used only in DWP, MWR, ISR, and ITL. Glucose-6-Phosphate was used in all sample types, although in a lesser degree in the Talik lake and the DH-GAP04 samples. The amino sugars N-acetyl-D-galactosamine and *N*-acetyl-D-glucosamine were used to some degree in all sample types, but not in the Talik lake. *N*-acetyl-D-glucosamine was also hydrolyzed anaerobically in DWP and ITL.

**FIGURE 6:**
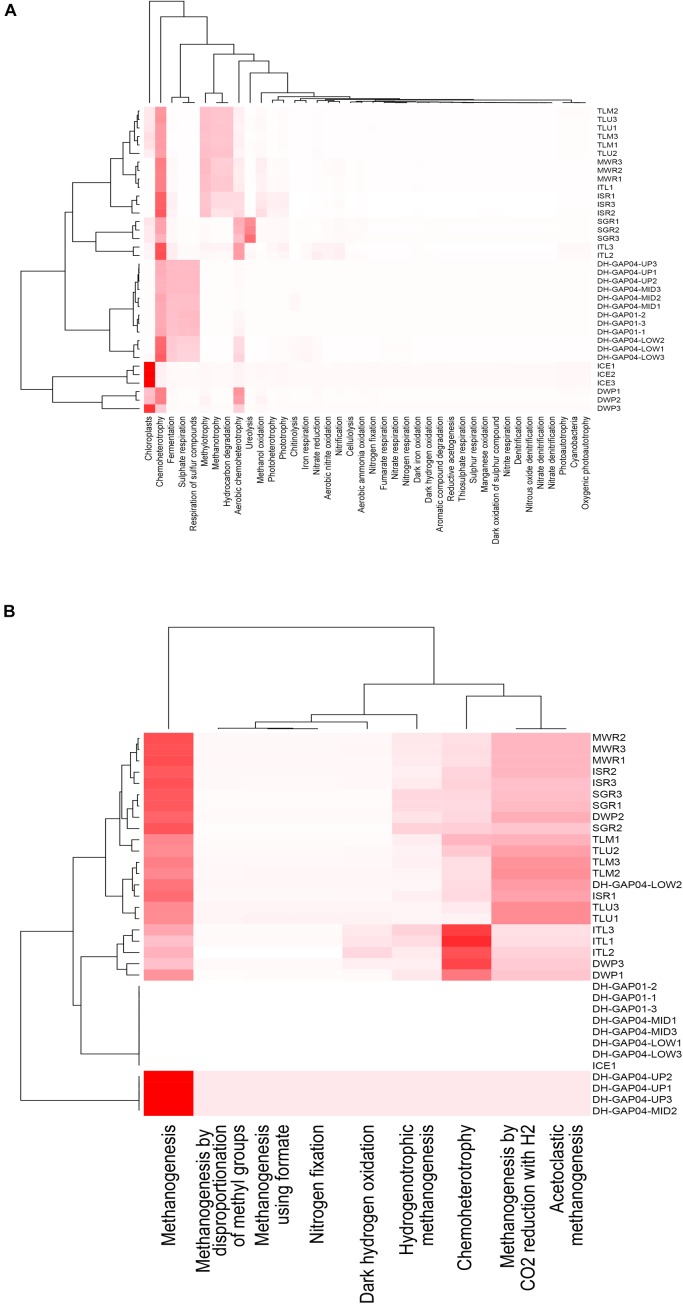
Heatmap cluster based on the relative functional abundance predictions of the **(A)** bacterial and **(B)** archaeal communities based on FAPROTAX. The cladograms were calculated using the Bray-Curtis dissimilarity model in phyloseq.

The FAPTOTAX predictions showed that chemoheterotrophy was the most common metabolic strategy in all identified bacterial communities, with the exception of the ICE, where chloroplasts and photosynthesis was most common (Figure 6A and Supplementary Table S2). Fermentation, sulfate and sulfur respiration were also prominent in the deep subsurface communities, whereas methylotrophy, methanotrophy and hydrocarbon degradation were prominent metabolic strategies in the Talik lake (TLU and TLM) communities and in the melt water communities (MWR, ISR, and ITL). Ureolysis, in addition to chemoheterotrophy, was the most prominent metabolic strategy in the SGR, although this was not seen in the ICE or the MWR samples. In the archaeal communities, aceticlastic and CO_2_-reducing-H_2_-oxidizing methanogenesis was the most common predicted metabolic strategy (Figure 6B and Supplementary Table S3). However, in the DWP and ITL, chemoheterotrophy was more prominent in the archaeal communities than methanogenesis.

## Discussion

We discovered a surprisingly wide microbial diversity in the permanently cold aquatic environments of western Greenland, ranging from glacier ice and melt water to river, lake and permafrost active layer melt water and deep groundwater from below the permafrost (Figure 1). The microbial communities clustered into 3 – 4 more or less distinct community types according to the different habitat types, namely lake/pond (DWP, TLU, and TLM), deep groundwater (DH-GAP01 and DH-GAP04 samples) and the melt water samples (SGR, MWR, and ISR) with ICE and ITL often grouping with different samples, depending on the analysis (Figure 4). ? showed that the bacterial communities in the inflow, lake water, and outflow of a lake system differed significantly from each other. The authors suggested that the environmental parameters and the prevailing microbial communities had a greater role in determining the microbial community composition than the input of new organisms from inflow water. It has been shown that the deep subsurface water of the Kangarlussuaq area of Greenland originates from glacier melt water (Claesson Liljedahl et al., 2016). However, the microbial communities inhabiting the deep groundwater differ greatly in composition from the communities in the water above the permafrost layer.

### Lake/Pond

#### Bacteria

The lake/pond samples (DWP, TLU, and TLM) were mostly affected by the concentration of DIC and the alkalinity (Figure 4A). Controversially, they contained high proportions, between 50.7 and 72.7%, of usually nitrogen-fixing Actinomycetes, such as *Planktophila*, typical in alkaline lakes (Jezbera et al., 2009) and *Frankiales*. According to the FAPROTAX analysis, these bacteria are responsible for the chemoheterotrophy in the lake. Bacteroidetes were also abundant in these samples, which also contribute to the chemoheterotrophy predicted in the bacterial communities (Figure 6A and Supplementary Table S2). *Flavobacteria*-like bacteria (also belonging to the Bacteroidetes) generally co-occur with periods of high heterotrophic activity and growth, and they have the ability to adapt to high-nutrient conditions (Newton and McMahon, 2011). Such conditions may have been prevailing in the lake ecosystem at the time of our sampling campaign, as we visited the site at the end of the growth season. Nitrogen fixation was not predicted by the FAPROTAX analysis for the Actinomycetes or Bacteroidetes phyla, but whole genome sequences of species belonging to the phylum Bacteroidetes (Inoue et al., 2015) have contained genes for nitrogen fixation.

Cyanobacteria were present in all lake/pond samples, but in the DWP their relative abundance was especially high. The Cyanobacteria were mostly similar to chloroplasts, indicating the importance of phytoplankton as primary producers in this ecosystem. The Talik lake samples, TLU and TLM, also contained a high relative abundance of methane-oxidizing Verrucomicrobia belonging to the novel *Methylacidiphilum* genus (Khadem et al., 2012), which also fixes nitrogen. The known *Methylacidiphilum* strains are extremely acidophilic, growing at pH as low as 1. However, in our study, approximately 12% of the bacterial sequences detected in the Talik lake samples (TLU and TLM) belonged to this genus, indicating a broader pH tolerance by this genus, or the presence of a novel type of *Methylacidiphilum.* This was also supported by the FAPROTAX predictions, which identified the *Methylacidiphilum* as both a methanotroph and a methylotroph (Figure 6A and Supplementary Table S2).

#### Archaea

The archaeal communities in the lake/pond samples were most affected by the concentration of organic carbon (NPOC), total N, inorganic carbon (DIC and HCO_3_) and alkalinity (Figure 4B). Woesearchaeota were the most prominent archaeal lineage present in the lake/pond (DWP, TLU, and TLM) samples. This novel archaeal phylum was first detected in deep sea hydrothermal vents (DHVEG-6, Takai and Horikoshi, 1999), but has since then been shown to be wide spread in aquatic environments. Woesearchaeota were for example found to be frequent and abundant archaeal inhabitants of oligotrophic, high-altitude lake water in the Pyrenees (Ortiz-Alvarez and Casamayor, 2016). Ortiz-Alvarez and Casamayor (2016) also suggested that the Woesearchaeota preferred environmental conditions that also promoted high bacterial diversity, which might explain why the organic carbon, total N and inorganic carbon concentrations affected the archaeal community in this habitat. Interestingly, the Woesearchaea live in aquatic habitats covering a pH range of at least 4.4 – 10.1 as reported by Ortiz-Alvarez and Casamayor (2016). Castelle et al. (2015) showed in a study of single cell genome re-construction that the Woesearchaea have very small genomes completely lacking genes for several common metabolic pathways, such as glycolysis/gluconeogenesis, and appears to have only partial pathways for the biosynthesis of nucleotides and amino acids. It has been proposed that this group of archaea live symbiotic/parasitic lives. Between 33.4 and 40.4% of the archaeal sequences in the lake/pond samples belonged to yet unidentified archaea. Due to novel high throughput sample handling and sequencing techniques the number of genomes of uncultured archaeal lineages are expanding almost exponentially (?). Thus, the genes contained in these new genomes as well as the functions and metabolic properties or phylogenetic affiliations of the genomes of the uncultured archaea may not yet be defined, which is reflected in the databases. Due to their novelty, Woesearchaea have not yet been included in the latest version of the FAPROTAX database.

#### Fungi

The fungal community did not appear to be affected by organic or inorganic carbon concentrations, nor by nitrogen concentration, only by salinity and sulfate concentrations (Figure 4C). The main fungal component in the lake/pond (DWP, TLU, and TLM) samples belonged to the zoospore-forming Chytridiomycota. These fungi are typical aquatic inhabitants and are important decomposers of recalcitrant material, thus contributing to the nutrient cycle by releasing more easily digestible compounds from e.g., plant cell walls to the general microbial community. In addition, the DWP contained a high proportion of saprobic *Ramicandelaber* fungi belonging to the Zygomycetes phylum (Ogawa et al., 2001). These fungi were quite recently described and are generally found in soil, but now also in aquatic habitats. It is possible that the DWP, being small and shallow, is more profoundly influenced by the surrounding soil than the Talik lake, thus having a surplus of Ramicandelaber. However, the active layer melt water infiltrating into the Talik lake (ITL) did not have a high relative abundance of these fungi, which suggests that the *Ramicandelaber* is not originating from the soil. Or, it may also be possible that the soil surrounding the DWP differs from the soil in the vicinity of the Talik lake. The Talik lake environment have been glacier free for longer than the DWP, which may affect the chemical composition of the soil, which could be less favorable for the Ramicandelaber fungi compared to the younger soil surrounding the DWP.

The lake/pond samples had the highest concentration of bacterial 16S rRNA and fungal 5.8S rRNA genes but the lowest cfu concentrations. The TLU and TLM also showed among the lowest number of utilized substrates in the Biolog AM tests.

### Deep Subsurface Environment

#### Bacteria

The bacterial community of the deep groundwater (DH-GAP01, DH-GAP04.up/.mid/.low) consisted mostly of Firmicutes (37.2 – 94.3%), which in contrast were almost absent in the surface water samples. The *Desulfosporosinus* genus had the highest relative abundance, with exception of DH-GAP04.low, contributing with up to 61% of the bacterial 16S rRNA gene sequences in these samples. The other significant firmicutes group belonging to the SRB2 cluster of the Thermoanaerobacterales family, contributed with 13.3 – 37.2% of the bacterial sequences in DH-GAP04 samples, but was absent in DH-GAP01. *Desulfosporosinus* species are known sulfate reducers with the capacity to also reduce Fe(III), nitrate, elemental sulfur ant thiosulfate (Pester et al., 2012; Sánchez-Andrea et al., 2015). The Thermoanaerobacterales SRB2 cluster is also highly likely sulfate reducers, although many Thermoanaerobacterales species do not reduce sulfate (e.g., Slobodkina et al., 2017). *Desulfosporosinus* species also have a broad range of possible electron donors and may even turn to fermentation in the absence of reducible electron acceptors (Sánchez-Andrea et al., 2015). The FAPROTAX predictions supported this as the *Desulfosporosinus* were the predominant bacteria deemed capable of fermentation, sulfate and sulfur compound respiration (Figure 6A and Supplementary Table S2). This makes the *Desulfosporosinus* very versatile bacteria able to survive in the harsh conditions of the arctic deep subsurface. Nixon et al. (2017) showed that *Desulfosporosinus* were important iron reducers in sub glacial arctic sediments, and this taxon appears to be wide spread in arctic anaerobic environments around the globe (Blanco et al., 2012). However, the FAPROTAX predictions did not show iron reduction for the *Desulfosporosinus*, neither did the deep subsurface community appear to be influenced by iron (Figure 4A). Instead, the samples containing the highest relative abundance of Desulfosporosinus were also most affected by the concentration of sulfate and total Sulfur.

#### Archaea

The archaea in the deep subsurface environments contributed with approximately 1% of the 16S rRNA gene pool. The main archaeal groups detected were the Ianarchaeum (DH-GAP01, DH-GAP04.mid), the Woesearchaeota (mainly DH-GAP04.up/.mid/.low) and the Bathyarchaeota (mainly DH-GAP04.mid). Ianarchaea, like the Woesearchaea, are minimal microbial cells dependent on other microbial species to support their growth (Golyshina et al., 2017). Nanorchaeota have been shown to grow together with specific *Thermoplasma* species in samples and cultures derived from acidic streamers, but in the deep Greenlandic subsurface groundwater the Nanorchaeota did not co-occur with any *Thermoplasma*. Due to the low abundance of archaea in these habitats, it is possible that these interactions were not seen because we worked close to the detection limit of the archaea. Bathyarchaeota, found in the DH-GAP04.mid and DH-GAP04.low samples have recently been described as a new archaeal phylum, formerly known as the Miscellaneous Crenarchaeotic Group, MCG (Evans et al., 2015). Based on single cell genome analyses this group of archaea have the necessary genes for methanogenesis, revealing that methanogenesis may be more widely spread among archaeal groups than previously thought. In addition, the fact that these archaea live in deep groundwater with an ambient temperature of 0–2°C may also suggest psychrophilic methanogenesis by these archaea.

#### Fungi

The fungal community in the deep subsurface samples was almost negligent. It should be considered, however, that the qPCR standard used for the estimation of fungal biomass is based on number of spores, and that spores may have several rRNA gene operons per genome. Nevertheless, the fungal communities were most affected by salinity and sulfate concentrations (Figure 4C).

*Cladosporium* was present in all samples (5.9 – 12.0 %), except DH-GAP04.low. This fungus is commonly found as a constituent of indoor mold communities and as parasites and pathogens of fungi and plants, respectively. Because the fungal community in the deep subsurface samples were very scarce, it is possible that the *Cladosporium* sequences in these samples are derived from contaminants. Nevertheless, these samples also contained different kinds of fungi previously detected in arctic environments, such as the Helotiales (Walker et al., 2011), Phaeococcomyces black yeast growing on rock surfaces (Gadd, 2007) and a variety of decomposer fungi (Singh and Singh, 2012). Despite their low abundance in the deep subsurface water, these fungi may be important decomposers in biofilms on rock surfaces in the subsurface environments. In addition, fungi weather rock releasing important nutrients for the use by the rest of the microbial community. Fossils of filamentous fungal structures have recently been revealed from deep subsurface rock (Bengtson et al., 2017). The heterotrophic anaerobic fungi inhabiting the anoxic deep subsurface release hydrogen through their metabolism, thus also supporting other microbial growth (Drake and Ivarsson, 2018). It has also been shown in igneous crystalline deep rock environments that there is a coupling between the anaerobic, rock-weathering fungi and sulfate reducing bacteria (Drake et al., 2017).

The deep subsurface waters had (together with ICE and SGR) the lowest number of bacterial and archaeal 16S rRNA and fungal 5.8S rRNA genes in this study, with the exception of the number of archaea in DH-GAP04.up and DH-GAP04.mid, where the archaeal number was approximately the same as in ISR and ITL. In contrast to the low gene copy numbers, the highest numbers of bacterial colonies were detected in DH-GAP04.up and DH-GAP04.mid, despite these samples being anoxic. The microbial communities hydrolyzed between 33 and 53 of the 95 substrates provided on the Biolog plate, similarly to the lake/pond communities. In addition to the sulfate reducing bacteria and the fastidious archaea, a subpopulation of heterotrophic aerobic bacteria inhabited this ecosystem.

### Ice and Melt Water

#### Bacteria

The glacial ICE had a surprisingly high number of bacterial 16S rRNA gene copies, 4.8 × 10^4^ mL^-1^. The population consisted mostly of Cyanobacteria, more exactly to Chloroplasts, which may originate from ice algae. Tanaka et al. (2016) found a large population of green algae and cyanobacteria in Siberian glaciers and showed that the biomass of algae increased with increasing temperature. Algae and cyanobacteria are primary producers, i.e., produce carbon compounds in their photosynthesis. In addition, these microorganisms fix atmospheric nitrogen. The produced carbon and nitrogen compounds are released in to the melt water and may be carried long distances from the point of primary production. Kohlbach et al. (2016) showed that the substrates produced by ice algae sustain ecological (macrobiota) key species in the Arctic Ocean, where the ice algae contribute with up to 25% of the primary produced carbon. This carbon strongly affects food webs in this ecosystem. In our study, the effect may be seen in the increase of microbial 16S rRNA gene copy numbers in the MWR and ISR, which are down flow from the glacier.

Proteobacteria was the most abundant bacterial phylum in the SGR, MWR and ISR, with beta- and gammaproteobacterial types as the most common. Psychrotrophic *Polaromonas* (1.1 – 7.4%) (Mattes et al., 2008; Darcy et al., 2011) and iron and manganese reducing *Albidiferax* (4.8 – 9.0%) (Lu et al., 2013; Akob et al., 2014; Kaden et al., 2014) were detected in all samples. The MWR and ISR also contained iron reducers belonging to the *Rhodoferax* (1.2 – 4.2%) (Kaden et al., 2014), some of which have been shown to be psychrotolerant and facultatively anaerobic (Finneran et al., 2003). This is well in agreement with the chemical parameters, as the bacterial communities in MWR, ISR and ITL were most strongly affected by the concentration of iron (Figure 4A). The *Rhodoferax* also contributed the majority of the phototrophy predicted to be present in the ISR and ITL (Figure 6A and Supplementary Table S2). Interestingly, the MWR and the ISR had a high relative abundance of methylotrophic bacteria belonging to the *Methylotenera* (6.3 – 8.8%) and *Methylobacter* (10.7 – 17.0%). *Methylobacter* species frequently been detected in arctic environments (Wartiainen et al., 2006). Although generally aerobic, *Methylobacter* species have been shown to perform anaerobic methane oxidation in sub-arctic lake sediments and to closely associate with iron reducers (Martinez-Cruz et al., 2017) and *Methylotenera* have also been shown to perform methane oxidation in arctic lake sediment (He et al., 2012). In addition, ISR had 5.4% of the 16S rRNA gene sequences belonging to *Crenotrix*, which have recently been shown to oxidize methane aerobically, but also anaerobically while reducing nitrate (Oswald et al., 2017). The FAPROTAX predictions also identified the *Methylotenera*, *Methylobacter*, and *Crenotrix* as the main methylotrophs in these samples (Figure 6A and Supplementary Table S2). *Undibacterium*, a genus ubiquitously found in aquatic (Chen et al., 2017) and soil (Li et al., 2017) environments was detected at high relative abundance (20.6%) only in SGR. *Massilia*, which contributed with a significant portion (12.7%) of the bacterial 16S rRNA gene pool in SGR, have been found in soil environments and especially as colonizers of plant roots (Ofek et al., 2012). *Massilia* could also play a significant role in the cycling of nitrogen compounds in the melting surface environment on glaciers, as they were predicted to be the main ureolytic microorganisms in the SGR (Figure 6A and Supplementary Table S2). Urea may be released from the decomposition of nitrogenous organic matter (Berman et al., 1999), which in this case could originate from the abundant Cyanobacteria found in the ICE. Our results indicate a more widespread distribution of habitats for this bacterium.

The MWR and ISR contained a high relative abundance of Parcubacteria (9.2 – 17.2%), which is a novel bacterial superphylum. These bacteria have reduced genome sizes, leading to limited metabolic capabilities, and they strongly rely on fermentation for their energy demand (León-Zayas et al., 2017). They are adapted to oxidative stress and they have genes for nitrate reduction. Due to their reduced metabolic capabilities in addition to the presence of genes for several adhesion and attachment proteins, it is assumed that they lead either symbiotic or parasitic lifestyles, but the assumption leans more toward symbiotic (Nelson and Stegen, 2015). The Bacteroidetes and Actinomycetes communities in these samples consisted of similar genera as in the lake/pond samples and were responsible for the chemoheterotrophic cycling of nutrients (Figure 6A and Supplementary Table S2). In the ITL samples, Parcubacteria dominated the bacterial communities with a relative abundance of up to 62.0%. The Proteobacteria were the second most abundant bacterial phylum, and also in ITL, *Methylobacter* contributed with 6.4% of the bacterial 16S rRNA gene sequences, indicating their important role in the oxidation of methane and methylated compounds in this environment (Figure 6A and Supplementary Table S2).

#### Archaea

The ICE had hardly any archaea according to the qPCR and only a few sequences from one ICE sample replicate, all belonging to the *Nitrosoarchaeum*, were obtained. The SGR had 2.5 × 10^2^ mL^-1^ archaeal 16S rRNA gene copies consisting almost exclusively of methanogens belonging to the *Methanosaeta* (37.3%) and *Methanospherula* (58.7%). The MWR had the highest abundance of Archaea measured in this study, with up to 1.2 × 10^4^ mL^-1^ archaeal 16S rRNA gene copies. The ISR had 1 order of magnitude less archaea, but the archaeal community structure in the MWR and ISR were very similar. The majority of the archaea belonged to the earlier mentioned Woesearchaeota. Methanogens belonging to the *Methanoregula* (17.5 – 19.1%) and *Methanosaeta* (3.3 – 5.1%) were also prominent. However, ammonia oxidizing *Nitrosoarchaeum* (Li et al., 2016) (5.9 – 8.2%) and anaerobic methane oxidizing *Methanoperedens* (formerly known as ANME-2D archaea) contributed with 5.2 – 7.3% of the archaeal 16S rRNA gene sequences. Putative methanogenic Bathyarchaeota (Evans et al., 2015) were also present in these samples (5.8 – 6.9%). This further implies that the methane and nitrogen cycling microbiota are important parts of the microbial communities of the melting arctic (Figure 6B and Supplementary Table S3). The ITL archaeal community was similar to that of the lake/pond samples.

#### Fungi

The fungal community in the ICE sample consisted of only 7.9 × 10^1^ mL^-1^ spore equivalents. In the SGR, MWR, ISR and ITL the fungal communities were over 10 times larger. Yeasts, such as *Cryptococcus* and *Rhodotorula* (Basidiomycetes) have previously been found in subglacial ice of high Arctic glaciers (Butinar et al., 2007), and the authors reported up to 4.0 × 10^3^ cfu mL^-1^ melt ice. Other yeasts, such as *Microbotryomyces*, were also found in these samples. Genera of the Glomeraceae were abundant and found exclusively in this group of samples. This phylum contains fungi that grow only in symbiotic mycorrhizal relationships with plants. They may have ended up in these environments as spores or sloughed off from plant roots from the surrounding terrestrial habitats. The rust fungus *Cronartium* was only detected in ITL, probably because the melt water from the active layer of the permafrost runs through shrub vegetated soil, from where the rust has infected the plants. Fungi belonging to the *Anaeromyces* were found only in the SGR, MWR, ISR, and ITL samples, but only at low relative abundances. Nevertheless, this fungus is obligately anaerobic (Breton et al., 1990), but in our samples found in aerobic habitats.

The number of cfu in the SGR, MWR, ISR and ITL samples were in the range of the deep subsurface samples, but the number of bacterial 16S rRNA gene copies and fungal 5.8S rRNA gene copies were 1 order of magnitude higher, with exception for the bacteria in the SGR. The ISR and ITL microbial communities hydrolyzed the highest number of substrates in the Biolog AM test, indicating a high metabolic variety of the microbial communities in these samples. The lack of autotrophically growing bacteria also support the dominance of heterotrophic strategies in the high Arctic aquatic systems.

## Summary

The microbial communities in the different habitat types differed both in taxonomical and metabolic characteristics. The most important microbial process in the lake/pond environment at the time of sampling was chemoheterotrophy. These heterotrophic bacteria have key positions in the degradation of organic matter and nutrient cycling in oligotrophic aquatic environments. Nitrogen fixation capacity was predicted to be low, according to the metabolic predictions, indicating that the bioavailable nitrogen is cycled through the breakdown of biomass, but may also originate from geological deposits in the host rock. The primary producing phytoplankton are also important key species because they provide photosynthetically produced carbon for the use of the rest of the microbial community. The relatively high abundance of fungi in the lake/pond environments also indicate that dead biomass needs to be recycled. Methane released from the anaerobic sediments in the lake/pond environment is oxidized by the methylotrophic Verrucomicrobia.

The deep subsurface biosphere functions mostly through sulfate or iron reduction or fermentation. Thus, the both the microbial community composition as well as the metabolic profiles of the communities in the deep subsurface differ from the microbial communities of the surface biosphere.

The algae of the glacial ice are important primary producers for the whole aquatic system as the ice melts. These algae release photosynthetically produced carbon compounds and nitrogen compounds from their nitrogen fixation, which is then transported away from the glacier. They may also function as a nitrogen source in the melt water through biomass breakdown. The melt water streams are rich in methane oxidizing bacterial and archaeal types, which may function together with iron reducing bacteria when oxidizing methane. The high relative abundance of methane oxidizers and iron reducers in the melt water streams may thus decrease the general methane emissions from arctic aquatic environments.

Most of the archaea and some of the bacteria detected in this study affiliated with newly described groups, which have extremely small genomes. It is still debated whether these microorganisms are symbionts or parasites. However, their high abundance in cold arctic aquatic environments may mean that they are symbionts. By serving as symbionts to larger microorganisms, these micro-archaea and –bacteria may provide their hosts with specific enhancement or advantage for better survival in these conditions. These small genome-size microorganisms appear to have in common the capacity for fermentation coupled with the release of hydrogen, which may aid the host.

Fungi and yeast degrade organic macromolecules in arctic conditions by secretion of cold-adapted enzymes. They also weather rock, which releases important mineral nutrients, and release hydrogen, which is a preferred energy source for many microorganisms. Thus, fungi contribute strongly to nutrient cycling in these environments.

## Conclusion

In conclusion, our results demonstrate that the different water types (lake/pond, deep subsurface, ice and melt water) differ from each other both by chemical and biological measurements, despite the fact that a great part of the waters originate from glacier melt water. The infiltration of water from one habitat type to the other, does not affect the microbial communities as much as the environmental conditions of the habitat do. For example, the harsh conditions in the subsurface below the permafrost does not support survival of most of the aerobic surface microorganisms. However, some cultivable aerobic microorganisms survive in the anaerobic deep subsurface, although in the deep subsurface conditions, they form a minority of the microbial community.

## Author Contributions

MB, AK, and TL conceived the study and performed the sampling. MB did the microbiology work and analyses. AK and TL were responsible for the chemistry data. LC gave valuable background information of the site and prepared the deep boreholes for sampling. MB wrote the manuscript. AK, TL, and LC commented on the manuscript.

## Conflict of Interest Statement

MB was employed by the VTT Technical Research Centre of Finland, Ltd., and received partial funding from the SKB and Posiva Oy during the study. LC was employed by the Svensk Kärnbränslehantering AB. TL and AK were employed by the Posiva Oy during the time of the study. The funding from the SKB and Posiva Oy was received in order to produce the manuscript. The authors declare that the research was conducted with highest academic integrity and without commercial interests.

## Footnotes

1 http://bccm.belspo.be/catalogs/lmg-media-details?CultureMediaID=72
