# Supplementary material for: Highly Diverse Aquatic Microbial Communities Separated by Permafrost in Greenland Show Distinct Features According to Environmental Niches
Source: Front Microbiol. 2019 Jul 11;10:1583. doi: 10.3389/fmicb.2019.01583 (PMC6637822; doi:10.3389/fmicb.2019.01583)
Supplement: Supplementary file 1 [file Data_Sheet_1.pdf]

Supplementary Material

1.1 Supplementary Figures

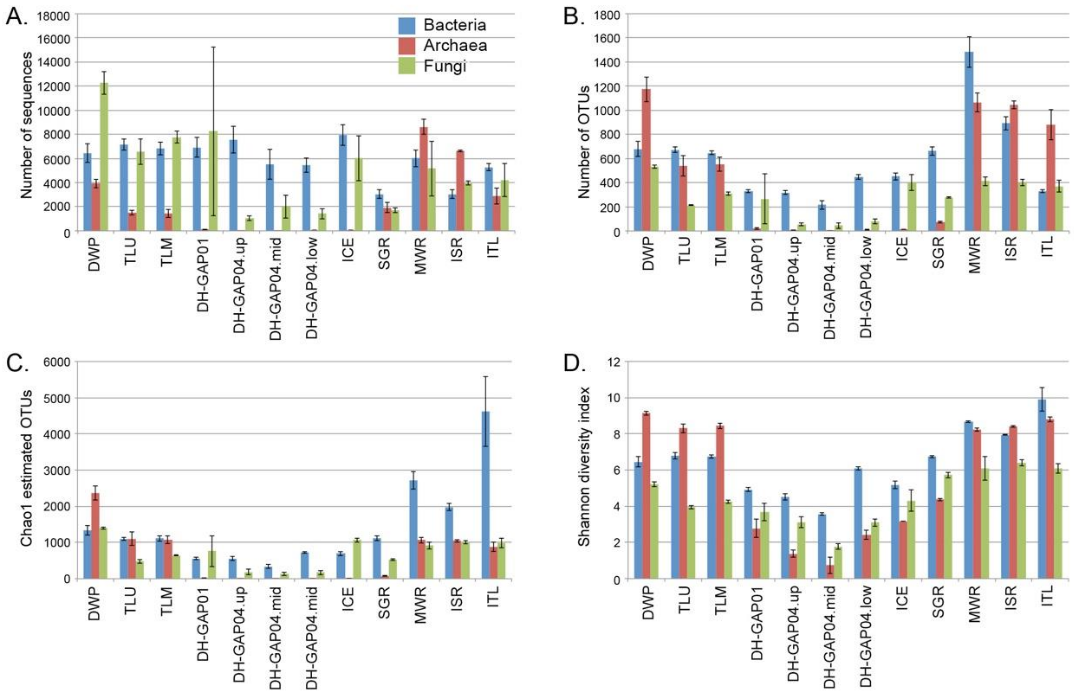

Supplementary Figure 1. The average number of A) sequence reads, B) OTUs, C) Chao1 estimated OTUs and D) Shannon diversity index for the bacterial (blue), archaeal (red) and fungal (green) sequence data. The mean values are calculated from 3 replicate amplicon libraries/sample and error bars indicate standard error of mean (SEM).

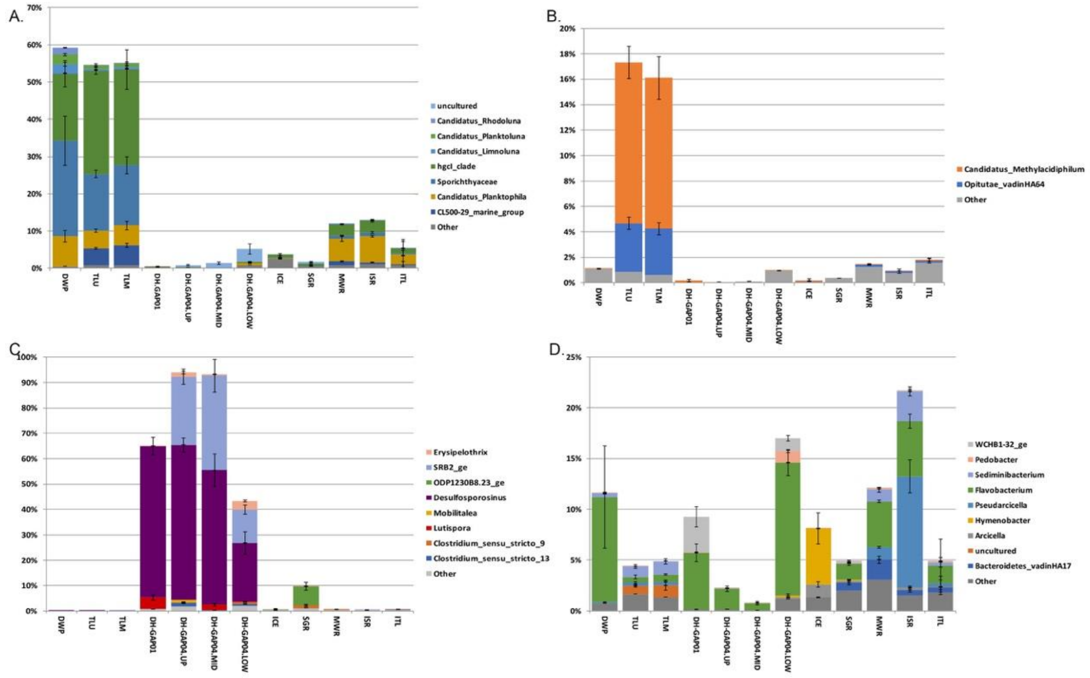

Supplementary Figure 2. The average relative abundance of genera belonging to A) the Actinobacteria, B) Verrucomicrobia, C) Firmicures and D) Bacteroidetes. The average values were calculated from 3 replicate amplicon libraries and the error bars in indicate SEM. Only genera contributing with at least 0.1% in any of the three replicate samples are shown individually. Groups contributing with lower relative abundance have been grouped together in the Other category.

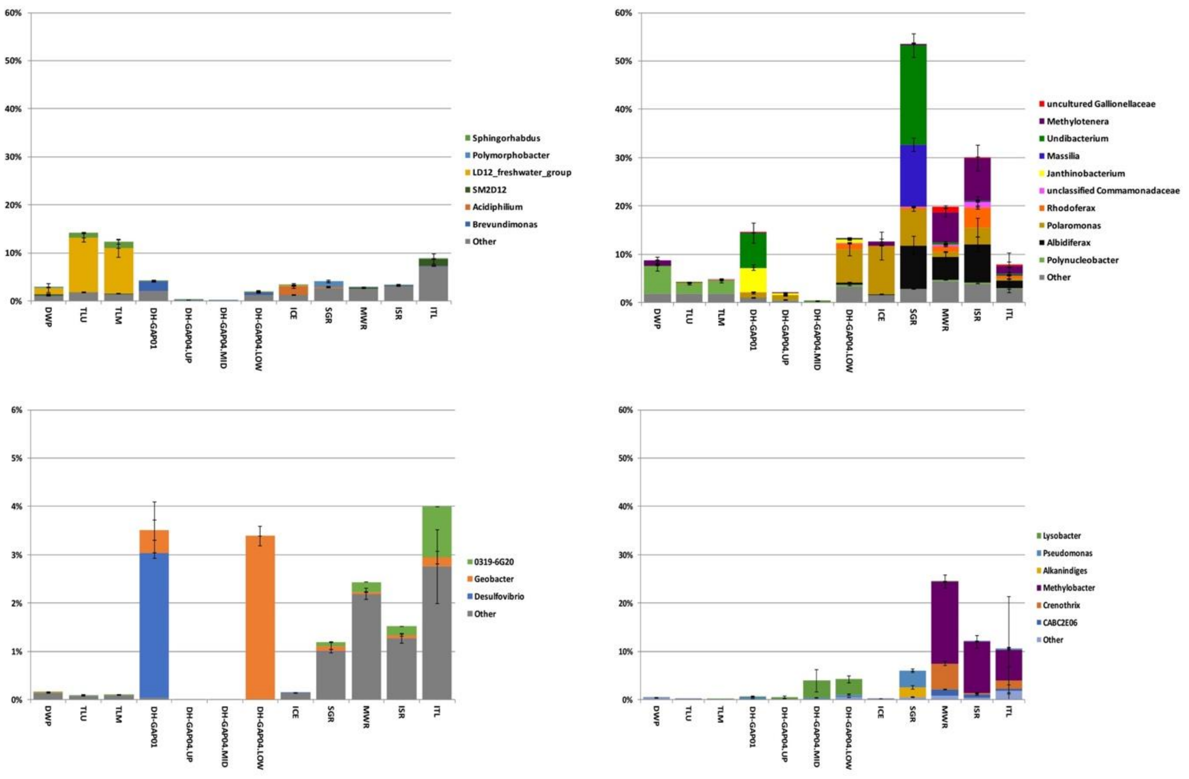

Supplementary Figure 3. The average relative abundance of proteobacterial genera belonging to A) Alphaproteobacteria, B) Betaproteobacteria, C) Deltaproteobacteria and D) Gammaproteobacteria. The average values were calculated from 3 replicate amplicon libraries and the error bars in indicate SEM. Only genera contributing with at least 0.1% in any of the three replicate samples are shown individually. Proteobacterial groups contributing with lower relative abundance have been grouped together in the Other category.

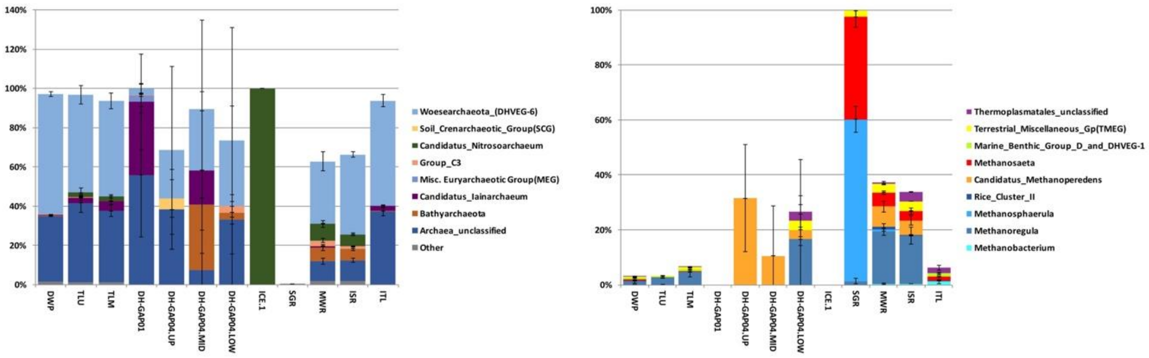

Supplementary Figure 4. The average relative abundance of archaeal genera belonging to A) the phyla Woesearchaeota, Thaumarchaeota, Diapherotrites, Bathyarchaeota and unclassified archaea, and, B) Euryarchaeota. The average values were calculated from 3 replicate amplicon libraries and the error bars in indicate SEM. Only genera contributing with at least 0.1% in any of the three replicate samples are shown individually. Archaeal groups contributing with lower relative abundance have been grouped together in the Other category in A.

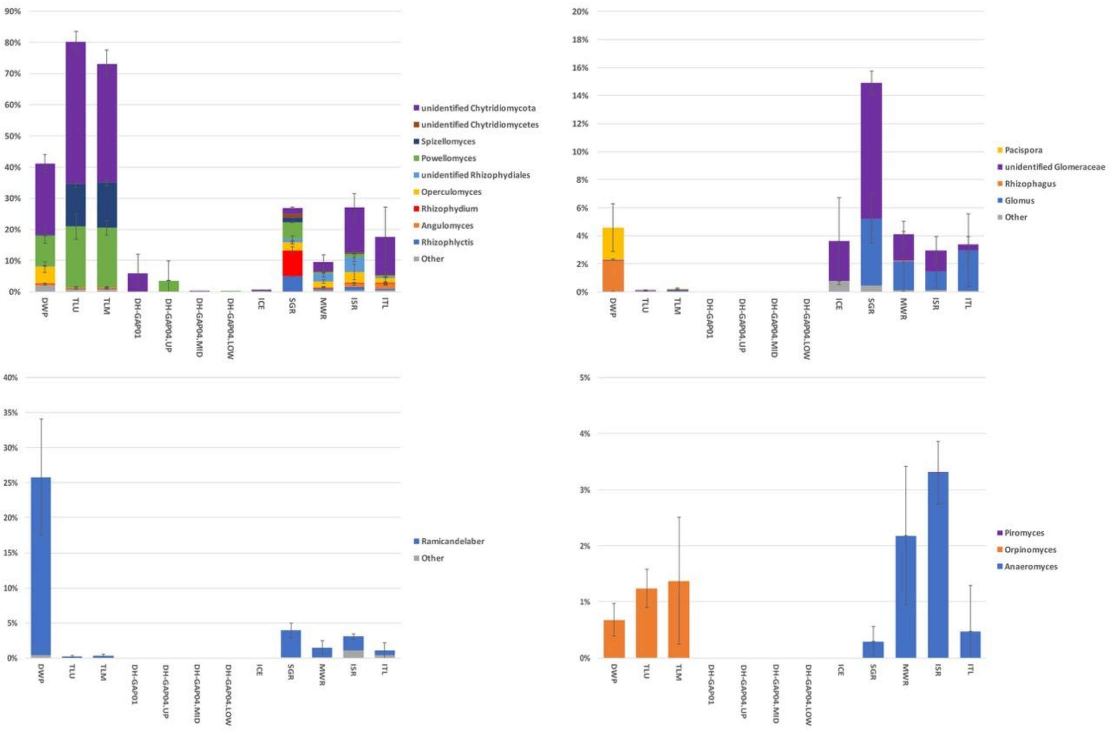

Supplementary Figure 5. The average relative abundance of fungal genera belonging to A) Chytridiomycota, B) Glomeromycota, C) Zygomycota and D) Neocallimastigomycota. The average values were calculated from 3 replicate amplicon libraries and the error bars in indicate SEM. Only genera contributing with at least 0.1% in any of the three replicate samples are shown individually. Groups contributing with lower relative abundance have been grouped together in the Other category.

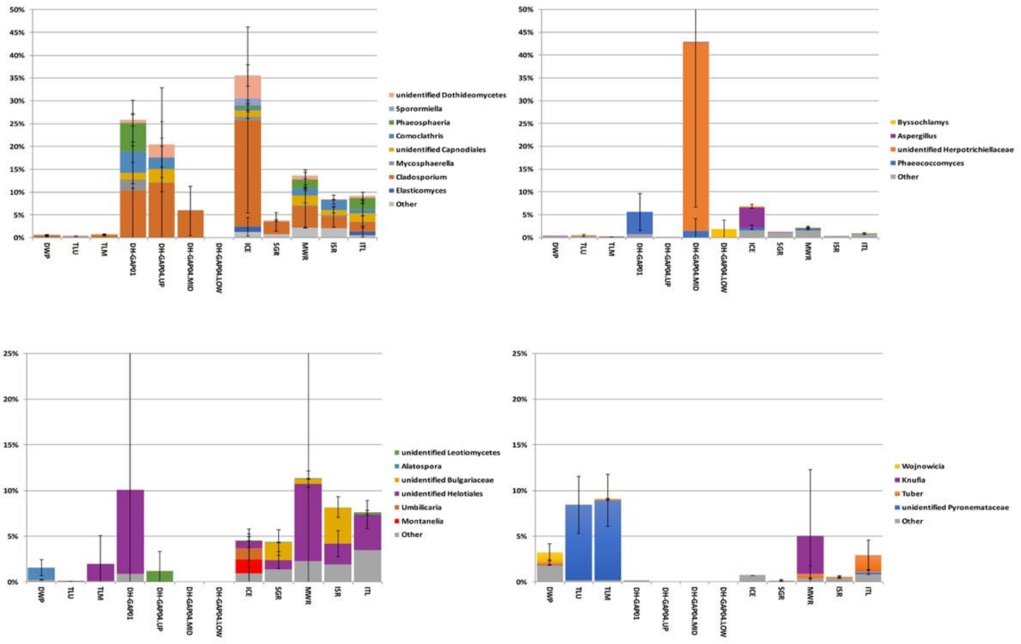

Supplementary Figure 6. The average relative abundance of genera of the Ascomycota belonging to A) Dothideomycetes, B) Eurotiomycetes, C) Leotiomycetes and D) Pezizomycetes. The average values were calculated from 3 replicate amplicon libraries and the error bars in indicate SEM. Only genera contributing with at least 0.1% in any of the three replicate samples are shown individually. Groups contributing with lower relative abundance have been grouped together in the Other category.

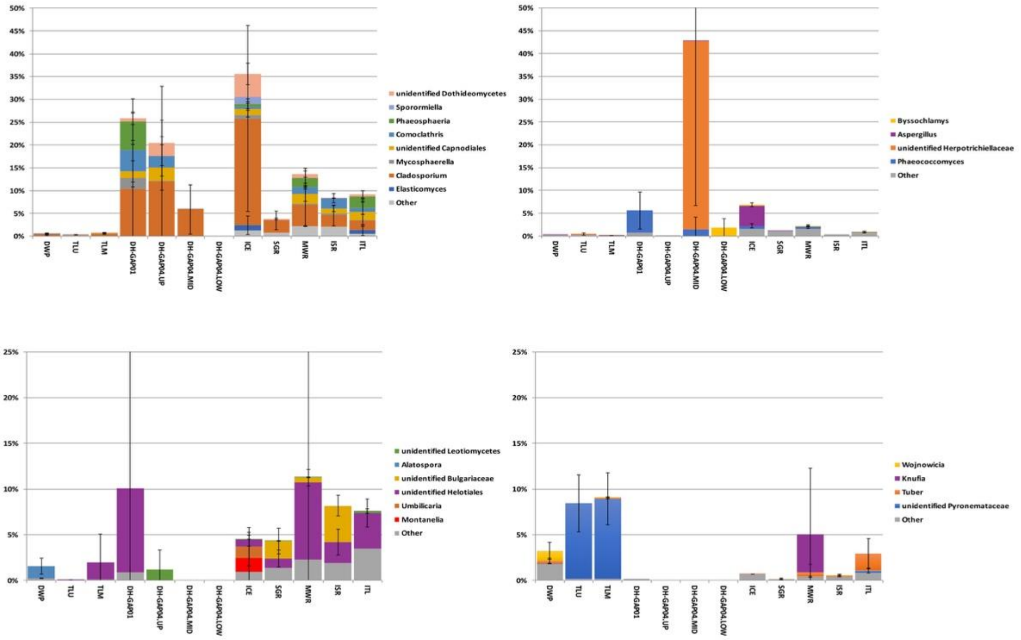

Supplementary Figure 7. The average relative abundance of genera of the Ascomycota belonging to A) Dothideomycetes, B) Eurotiomycetes, C) Leotiomycetes and D) Pezizomycetes. The average values were calculated from 3 replicate amplicon libraries and the error bars in indicate SEM. Only genera contributing with at least 0.1% in any of the three replicate samples are shown individually. Groups contributing with lower relative abundance have been grouped together in the Other category.
