# Supplementary material for: Highly Diverse Aquatic Microbial Communities Separated by Permafrost in Greenland Show Distinct Features According to Environmental Niches
Source: Front Microbiol. 2019 Jul 11;10:1583. doi: 10.3389/fmicb.2019.01583 (PMC6637822; doi:10.3389/fmicb.2019.01583)
Supplement: Supplementary file 2 [file Table_1.pdf]

Supplementary Material

Supplementary Table 1. The alphadiversity estimates of the samples; number of sequences (Sequences), number of OTUs (OTUs), Chao1 OTU number estimate (Chao1) and Shannon diversity index (Shannon). The values are means of 3 replicate amplicon libraries/sample. Standard error of mean (SEM) is indicated below each mean value.

		DWP	TLU	TLM	DH-GAP01	DH-GAP04.up	DH-GAP04.mid	DH-GAP04.low	ICE	SGR	MWR	ISR	ITL
Bacteria													
Sequences	Bacteria	6438	7143	6820	6917	7579	5537	5455	7947	3040	6009	3040	5279
	SEM	759	457	526	807	1122	1255	602	883	372	716	372	322
OTUs	Bacteria	680	673	647	329	318	216	448	452	662	1482	893	329
	SEM	64	24	14	11	17	36	19	29	35	126	55	11
Chao1	Bacteria	1337	1099	1109	556	557	341	718	692	1117	2722	1981	4617
	SEM	141	40	74	33	50	59	24	55	75	245	103	966
Shannon	Bacteria	6,4	6,8	6,7	4,9	4,5	3,6	6,1	5,2	6,7	8,7	7,9	9,9
	SEM	0,3	0,2	0,1	0,1	0,2	0,1	0,1	0,2	0,0	0,1	0,0	0,7
Archaea													
Sequences	Archaea	3928	1517	1425	112	12	10	29	41	1927	8622	6624	2877
	SEM	359	198	322	42	8	5	21	0	407	638	76	648
OTUs	Archaea	1174	539	554	18	3	2	9	13	73	1064	1046	881
	SEM	103	83	59	6	1	1	3	0	8	76	34	127
Chao1	Archaea	2367	1100	1078	18	3	2	9	13	73	1064	1046	881
	SEM	191	183	105	6	1	1	3	0	8	76	34	127
Shannon	Archaea	9,1	8,3	8,4	2,8	1,4	0,7	2,4	3,2	4,4	8,2	8,4	8,8
	SEM	0,1	0,3	0,1	0,5	0,2	0,5	0,3	0,0	0,1	0,1	0,0	0,1
Fungi													
Sequences	Fungi	12257	6577	7765	8248	1033	2014	1427	6016	1695	5159	3958	4206
	SEM	926	1059	495	6993	190	953	432	1857	178	2268	163	1361
OTUs	Fungi	532	214	307	266	55	44	79	401	278	411	401	369
	SEM	13	3	13	209	12	22	22	66	5	38	28	49
Chao1	Fungi	1396	477	640	761	184	134	169	1066	519	921	1000	992
	SEM	27	49	10	425	79	53	61	54	22	99	43	137
Shannon	Fungi	5,2	3,9	4,3	3,7	3,1	1,8	3,1	4,3	5,7	6,1	6,4	6,1
	SEM	0,1	0,1	0,1	0,5	0,3	0,2	0,2	0,6	0,2	0,6	0,2	0,3
